# Calcium Silicate-Based Cements in Restorative Dentistry: Vital Pulp Therapy Clinical, Radiographic, and Histological Outcomes on Deciduous and Permanent Dentition—A Systematic Review and Meta-Analysis

**DOI:** 10.3390/ma17174264

**Published:** 2024-08-28

**Authors:** Maria Teresa Xavier, Ana Luísa Costa, João Carlos Ramos, João Caramês, Duarte Marques, Jorge N. R. Martins

**Affiliations:** 1 Centre for Innovation and Research in Oral Sciences (CIROS), Institute of Paediatric and Preventive Dentistry, Faculty of Medicine, University of Coimbra, 3000-075 Coimbra, Portugal; 2 Instituto de Implantologia, 1070-064 Lisboa, Portugal; carames@campus.ul.pt (J.C.); duarte.marques@campus.ul.pt (D.M.); 3 Laboratory of Biomechanical tests and Centre for Innovation and Research in Oral Sciences (CIROS), Institute of Operative and Preventive Dentistry, Faculty of Medicine, University of Coimbra, 3000-075 Coimbra, Portugal; joao.ramos@ipmd.pt; 4 Faculdade de Medicina Dentária, Universidade de Lisboa, 1600-277 Lisboa, Portugal; 5 LIBPhys-FCT UID/FIS/04559/2013, 1600-277 Lisboa, Portugal; 6 Centro de Estudos de Medicina Dentária Baseada na Evidência (CEMDBE), 1600-277 Lisboa, Portugal; 7 Grupo de Investigação em Bioquímica e Biologia Oral (GIBBO), Unidade de Investigação em Ciências Orais e Biomédicas (UICOB), 1600-277 Lisboa, Portugal

**Keywords:** calcium silicate-based cements, permanent teeth, primary teeth, review, vital pulp therapy

## Abstract

Vital pulp therapy aims to preserve the vitality of dental pulp exposed due to caries, trauma, or restorative procedures. The aim of the present review was to evaluate the clinical, radiographic, and histological outcomes of different calcium silicate-based cements used in vital pulp therapy for both primary and permanent teeth. The review included 40 randomized controlled trials from a search across PubMed, LILACS, and the Cochrane Collaboration, as well as manual searches and author inquiries according to specific inclusion and exclusion criteria. A critical assessment of studies was conducted, and after data extraction the results were submitted to a quantitative statistical analysis using meta-analysis. The studies, involving 1701 patients and 3168 teeth, compared a total of 18 different calcium silicate-based cements in both dentitions. The qualitative synthesis showed no significant differences in short-term outcomes (up to 6 months) between different calcium silicate-based cements in primary teeth. ProRoot MTA and Biodentine showed similar clinical and radiographic success rates at 6 and 12 months. In permanent teeth, although the global results appeared to be well balanced, ProRoot MTA generally seemed to perform better than other calcium silicate-based cements except for Biodentine, which had comparable or superior results at 6 months. Meta-analyses for selected comparisons showed no significant differences in clinical and radiographic outcomes between ProRoot MTA and Biodentine over follow-up periods. The present review highlights the need for standardized definitions of success and follow-up periods in future studies to better guide clinical decisions. Despite the introduction of new calcium silicate-based cements aiming to address limitations of the original MTA. ProRoot MTA and Biodentine remain the most used and reliable materials for vital pulp therapy, although the results did not deviate that much from the other calcium silicate-based cements. Further long-term studies are required to establish the optimal CSC for each clinical scenario in both dentitions.

## 1. Introduction

The primary aim of vital pulp therapy is to preserve the vitality of the dental pulp when it is exposed due to caries excavation, trauma, restorative procedures, or anatomical anomalies. The principles underlying this treatment are based on cellular mechanisms involved in pulp repair and bridge formation, which create an environment that induces hard tissue formation by the remaining pulp cells, seals the exposure site and contributes to sustained pulp vitality [1,2]. Bacterial contamination, resulting from pulp tissue exposure, promotes an immune response followed by cell recruitment from the dentin–pulp complex and hard tissue formation by differentiated progenitor cells, leading to reparative dentin formation [3]. In clinical practice, pulp capping materials are placed between the vital pulp and the external environment to protect the pulp and the exposure site [1,2]. Minor indirect trauma to pulp tissue without exposure stimulates existing primary odontoblasts to produce reactionary dentin. Therefore, materials used in vital pulp treatments should adhere to biological principles, such as adequate biocompatibility and bioactivity, and consequently promote dental pulp stem cell activity and pulp healing in permanent teeth [4].

In short, vital pulp therapy techniques include various definitive options for both primary and permanent teeth. These options, from least to most invasive, are direct pulp capping [5], partial pulpotomy [6,7], and full/coronal pulpotomy. Ultimately, treatment selection depends on the extent of the remaining healthy pulp tissue and the size of the exposure [5,8].

The optimal endodontic material, which can guarantee long-term treatment success, should include the following characteristics: biocompatibility, radiopacity, antibacterial properties, dimensional stability, ease of handling, resistance to blood contamination and dislodging forces, the ability to set in a wet environment, and hard-tissue conductivity [9,10]. Additionally, it should block the communication pathways of bacteria and fluids between the root canal system and adjacent dental tissues [11,12] to ensure a biological seal, stimulate hard tissue production, and induce tissue repair [13,14]. Many materials have been used (or suggested for use), including adhesives, calcium hydroxide, or calcium silicate-based cements [15]. Nowadays, it is commonly accepted that the last group is the gold standard for vital pulp therapy procedures.

Mineral Trioxide Aggregate (MTA, Dentsply, Tulsa, OK, USA) was the first calcium silicate-based cement introduced into the market and has been widely studied for its effects on primary and permanent teeth [15]. MTA offers properties such as biocompatibility, antimicrobial effects [5,16,17,18], and the ability to maintain pulp integrity and physiological function [18,19]. Furthermore, it has been found capable of preventing infiltration, exhibiting good sealing ability [5,16,17,20], inducing hard tissue formation [5,21], and promoting tissue neoformation when placed in contact with dental pulp or periradicular tissues, without cytotoxic effects [18,22]. However, handling difficulties, long setting time, and tooth discoloration have been identified as its main limitations [17,22].

Currently, there are commercially available alternatives to the original MTA or new generations of calcium silicate-based cements. These include Angelus MTA (Londrina, Paraná, Brazil); Biodentine (Septodont, Saint-Maur-des-Fossés Cedex, France); CEM (BioniquDent, Tehran, Iran); Endocem (Endocem, Maruchi Regenerative Endodontic Materials); Gray ProRoot MTA (Dentsply Tulsa Dental, Johnson City, TN, USA); iRoot BP (Innovative BioCeramix, Inc., Vancouver, BC, Canada); MTA Plus (Avalon Biomed Inc., Houston, TX, USA); NeoMTA Plus (Avalon Biomed Inc., Bradenton, FL, USA); OrthoMTA (BioMTA, Seoul, Republic of Korea); Portland cement (Votorantim Cimentos, São Paulo, SP, Brazil); Retro MTA (BioMTA, Seoul, Republic of Korea); TheraCal LC (Bisco Inc., Schaumburg, IL, USA); and White ProRoot MTA (Dentsply Tulsa Dental, Johnson City, TN, USA), among others. These products claim to overcome some of the limitations of the original MTA.

Considering the vast number of calcium silicate-based cements, specifically MTA-type products, that have been introduced into the market, extensive research has been carried out focusing on the effect of these materials on vital pulp treatment in both dentitions. A search in the PubMed database using the combination of terms (“teeth” AND “pulpotomy” AND “MTA”) and (“direct pulp capping” AND “MTA”) was able to identify 16 previous systematic reviews [15,23,24,25,26,27,28,29,30,31,32,33,34,35,36,37]. Although an extensive synthesis has already been conducted, it is important to note that many of these reviews focus on comparisons with other material families, such as calcium hydroxide in permanent dentition, or formocresol in deciduous dentition, rather than comparing calcium silicate-based cements to each other. Additionally, the basic studies that do compare these materials to each other present very distinct follow-up times or even define success at different levels (clinical, radiographic, or even histological). However, for clinicians, in addition to knowing which material family is better, it is important to know which specific calcium silicate-based cement is most recommended for each vital pulp treatment.

Considering the lack of a general consensus regarding follow-up periods, success outcomes, or their definitions in basic research studies comparing calcium silicate-based cements, and the fact that previous systematic reviews did not take that into account, the present systematic review was designed to determine if a preferred calcium silicate-based cement exists for each type of vital pulp treatment in both dentitions. This consideration takes into account the characteristics of dental tissues and the specifics of each treatment option (such as material thickness, extent of pulp exposure, and diagnosis) as well as the clinical situation (including patient age, cooperation, and isolation conditions).

The null hypothesis to be addressed in the present review was that there are no differences between different calcium silicate-based cements concerning clinical, radiographic, and histological outcomes when used for vital pulp therapy in permanent and deciduous dentitions.

## 2. Materials and Methods

### 2.1. Review Registration and Study Design

The protocol for this systematic review and meta-analysis was registered in the International Prospective Register of Systematic Reviews (PROSPERO) (CRD42020196232) and adhered to the Preferred Reporting Items for Systematic Reviews and Meta-Analyses (PRISMA) guidelines [38].

### 2.2. Search Strategy and Information Sources

Three electronic databases (PubMed, LILACS, and the Cochrane Collaboration) were accessed, and an electronic search was conducted for randomized controlled trials comparing the outcomes of at least two calcium silicate-based cements used in vital pulp therapies. Table 1 summarizes the terms and filters used for each database. A hand search was also performed by reviewing all reference lists from the previously identified studies and by searching in three relevant peer-reviewed scientific journals (*Pediatric Dentistry*, *Journal of Endodontics*, and *International Endodontic Journal*) as well as in previously published systematic reviews on the topic. Additionally, the authors of the identified studies were contacted via email and asked if they were aware of any additional information, including published articles, grey literature, or unpublished material.

### 2.3. Study Selection and Screening Process

The final selection of studies followed a three-stage assessment. Initially, the titles and abstracts of the scientific papers were screened and labeled as ‘relevant’ or ‘irrelevant’ according to predefined inclusion and exclusion criteria (Table 2) (stage 1). The studies considered relevant in the initial screening had their full texts analyzed and re-labeled using the same predefined criteria (stage 2). In the final stage, all selected papers underwent a critical appraisal of their scientific merit (stage 3). The review search was conducted between August 2019 and June 2021 and was updated in October 2023 and April 2024, considering all studies published from January 1990 until the update date. No language restrictions were applied.

### 2.4. Critical Appraisal

A critical appraisal of the selected studies was conducted using the Critical Appraisal Skills Programme (CASP) checklist for randomized controlled trials [39]. Each scientific paper was evaluated by two reviewers (T.X. and J.M.), who scored each CASP question as “yes,” “cannot tell,” or “no.” The results of both reviewers were compared to determine their agreement percentage, and any discrepancies were discussed until a final consensus was reached. The final score percentage for each paper was calculated based only on the consensual “yes” answers. The studies were categorized as having a “high” risk of bias (RoB) if they scored 49% or lower, “moderate” RoB if they scored between 50% and 69%, or “low” RoB if they scored 70% or higher [40]. According to the eligibility criteria (I-CASP) (Table 2), the studies with a high risk of bias were excluded from the review.

### 2.5. Data Items and Statistical Analysis

To conduct a proper qualitative synthesis, data regarding patient demographics, tooth groups, preoperative diagnosis, treatment performed, restorative crown filling materials and their placement timing, and clinical and radiographic success rates at available follow-up times were retrieved from the pooled studies and combined into review synthesis tables. A quantitative synthesis (meta-analysis) was performed on groups where the comparison between two specific calcium silicate-based cements was available in at least three different studies that presented an equivalent definition of success in the assessed parameters over the same follow-up period. Additionally, histological data, such as bridge formation and degree of inflammation, were also included in the qualitative synthesis.

The data from the pooled randomized controlled trials submitted for quantitative synthesis were processed using OpenMeta [Analyst] v. 10.10 software. The pooled results were presented as risk ratio (RR) forest plots using a random-effects model (DerSimonian–Laird test) with a 95% confidence interval (CI). Tau² (estimate of between-study variance) was used to assess heterogeneity among studies, while the Q-Cochran test with the DerSimonian and Laird method and the I² statistic were used to determine the statistical heterogeneity of outcomes. An I² value of 50% or higher was considered indicative of significant heterogeneity. Possible sources of heterogeneity were assessed by conducting meta-regression [41,42]. Statistical significance was set at *p* < 0.05.

## 3. Results

### 3.1. Selected Studies

**Figure 1:**
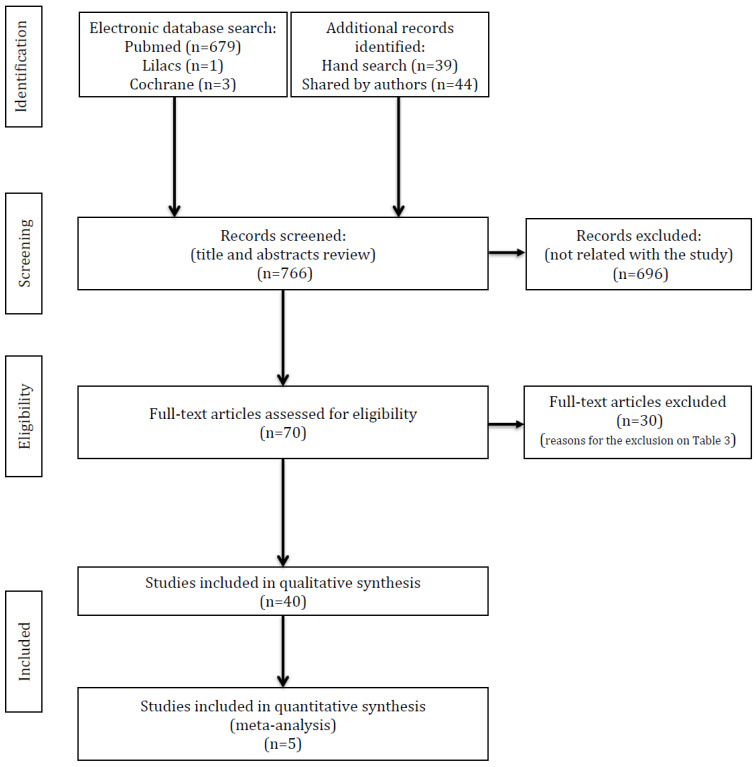
Flow diagram of the search strategy.

The search in electronic databases resulted in 683 references, while the manual search identified an additional 39. The response rate from authors was 40% (16 replies out of 40 contacts), resulting in 44 additional recommended studies. From the 766 references initially identified, after excluding duplicates and those that did not meet the inclusion criteria, 70 articles had their full texts screened. From these, a final pool of 40 randomized controlled trials was included in the present review for qualitative analysis, and five of these were also included in the meta-analysis (Figure 1). Reasons for excluding studies [43,44,45,46,47,48,49,50,51,52,53,54,55,56,57,58,59,60,61,62,63,64,65,66,67,68,69,70,71,72] after full-text analysis can be seen in Table 3. Regarding the quality assessment using the CASP questionnaire, the overall agreement between the two reviewers was considered good (Table 4), and the final average CASP score for the 40 included studies was 87%.

Of the 40 pooled studies, 22 were related to deciduous dentition [16,17,20,74,76,77,78,79,80,81,82,83,84,85,86,87,88,89,90,91,92,93] and 18 to permanent dentition [72,73,94,95,96,97,98,99,100,101,102,103,104,105,106,107,108,109]. A total of 1701 patients were involved (675 children and 614 adults), with the age of patients undetermined in 13 articles. Data from 3168 teeth were collected, which included 5 incisors, 174 premolars, and 1394 molars (one paper reported on 24 incisors and premolars without specifying the number of each group, and another reported 104 molars or premolars without specifying the number). For deciduous dentition, all teeth were molars (1467 teeth).

Regarding the pulp dressing material, the 40 included studies evaluated a total of 18 calcium silicate-based cements in both dentitions, with ProRoot MTA, Biodentine, and Angelus MTA being the most used. The number of studies using each specific pulp dressing material in deciduous dentition was as follows: ProRoot MTA (16 studies), Biodentine (7), Angelus MTA (6), CEM (2), Portland cement (3), MTA Plus (1), NeoMTA (2), Gray ProRoot MTA (1), TheraCal-LC (2), OrthoMTA (1), Retro MTA (1), Bio-C Pulpo (1), NeoPUTTY (1), BC Putty (1), and Endocem (1). For permanent dentition, the materials used were: ProRoot MTA (13 studies), Biodentine (9), Angelus MTA (2), Gray ProRoot MTA (2), Angelus MTA Gray (2), CEM (3), Retro MTA (2), iRoot BP (2), OrthoMTA (1), Endocem (1), TotalFill (1), MTA Plus (1), and TheraCal-LC (2). The evaluated outcomes in the 40 randomized controlled trials assessed vital pulp therapy according to three distinct parameters (clinical, radiographic, or histological), with follow-up periods ranging from 6 to 24 months for deciduous dentition and from 1.5 to 60 months for permanent dentition. Table 5, Table 6 and Table 7 summarize the success criteria definitions for each of the three outcome parameters, which, despite several similarities among studies, also presented specificities inherent to each study.

### 3.2. Deciduous Teeth

**Figure 2:**
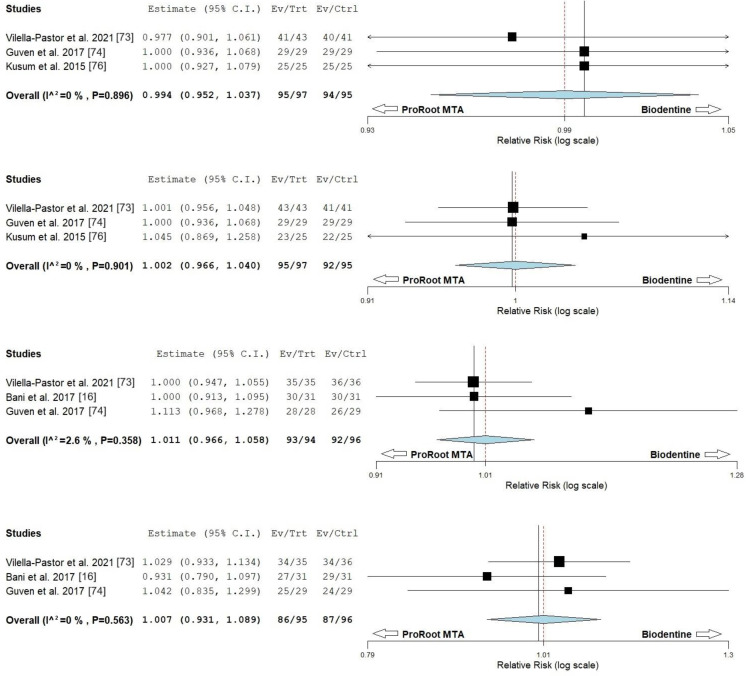
Risk ratio forest plots comparing the ProRoot MTA and Biodentine in deciduous dentition. From top to bottom: clinical success (6 months); radiographic success (6 months); clinical success (2 years); radiographic success (2 years). No significant differences were noted between both material outcomes the any of the follow-up periods.

The 22 studies focused on deciduous dentitions performed a total of 18 material comparison combinations (Table 8). The most tested combination was the comparison between ProRoot MTA and Biodentine (six studies), which revealed a very balanced success rate between them at various follow-up periods. ProRoot MTA showed clinical and radiographic outcomes above 96.8% and 86.2%, respectively, at a 2-year period, while Biodentine had outcomes above 89.7% and 82.8% during the same period. Four studies from this comparison could be included in the meta-analysis. The forest plots for the 6-month follow-up showed a clinical and radiographic relative risk of 0.994 (0.952–1.037) and 1.002 (0.966–1.040), respectively, with heterogeneity of τ² (0.00), χ² (0.220; df = 2), I² (0%) and τ² (0.00), χ² (0.209; df = 2), I² (0%), respectively (*p* > 0.05). For the 2-year follow-up, the clinical and radiographic relative risk was 1.011 (0.966–1.058) and 1.007 (0.931–1.089), with heterogeneity of τ² (0.00), χ² (2.053; df = 2), I² (2.6%) and τ² (0.00), χ² (1.150; df = 2), I² (0%), respectively (*p* > 0.05) (Figure 2). Two studies compared ProRoot MTA and CEM, with both materials showing success rates around 90% at 20 months and 2 years. Additionally, Angelus MTA and Portland cement were compared in two studies, with both showing a 100% clinical and radiographic success rate at 2 years. Both materials demonstrated dentin-like mineralization and dentin barrier formation at the time of tooth exfoliation, which ranged from 6 to 24 months after the vital pulp treatment (Table 9). A thick layer of dentin bridge formation was also observed when comparing White ProRoot MTA and Grey ProRoot MTA. Fifteen more pulp dressing material combinations were tested, with sample sizes ranging from 15 to 52 teeth per group and follow-ups from 6 months to 2 years (Table 8). Nine of these studies used ProRoot MTA, five tested either Angelus MTA or Biodentine, and one compared NeoMTA with NeoPUTTY. None of the combinations documented significant differences among the tested materials. An additional overview of these studies showed a tendency for slightly lower success percentages in the radiographic evaluation compared to the clinical evaluation. Due to the lack of studies, no more combinations were pooled into the meta-analysis.

### 3.3. Permanent Teeth

Nineteen material comparison combinations were conducted in the 18 studies addressing the permanent dentition response (Table 10). The most common comparison was between ProRoot MTA and Biodentine (six studies). Two of these studies provided very short outcome periods, 1.5 months and 2 months, both reporting a 100% success rate for both materials in clinical and radiographic assessments. Another study achieved the same outcome at a 1-year follow-up. Both materials were able to induce dentin bridge formation at 6 and 8 weeks, while presenting an equivalent level of pulp inflammation (Table 9). ProRoot MTA and Retro MTA were compared twice, with one clinical and radiographic evaluation showing a success rate of approximately 96% for both materials at a 1-year follow-up period. The longest follow-up period was in a study comparing ProRoot MTA and CEM, which noted a radiographic success rate of 84.7% and 78.1%, respectively, and approximately 98% clinical success for both materials at a 5-year control period. This same study was the only one to report a statistically significant difference among all the performed comparison combinations, with the radiographic outcome of ProRoot MTA (94.9%) being significantly higher than that of CEM (86.1%) at the 2-year follow-up period (*p* < 0.05) (Table 10). All the other 16 combinations present in single studies showed very well-balanced results in all assessed control periods. All comparisons demonstrated acceptable histological responses regarding bridge formation, although ProRoot MTA appeared to have a superior response compared to TheraCal-LC or Retro MTA (Table 9). Due to the limited number of studies, no pulp dressing material combinations tested on permanent teeth were pooled into the meta-analysis.

## 4. Discussion

The growing number of new patents and published studies on calcium silicate-based cements highlights the importance of this treatment option in vital pulp therapy. However, the optimal choice for each clinical scenario, whether in primary or permanent dentition, remains unclear in the literature. Therefore, this review seeks to offer high-quality evidence on the clinical, radiographic, and histological effectiveness of various calcium silicate-based cements used in vital pulp treatment. Different types of calcium silicate-based cements have been identified for each form of vital pulp therapy, which will be further explored to present their current trends and future outlooks.

In pulpotomy and direct pulp capping performed in primary dentition, there were no significant discrepancies between different calcium silicate-based cements. Specifically, there was no evidence to support different clinical and radiographic outcomes in the short term (up to 6 months) when comparing ProRoot MTA with other cements. Compared with Biodentine, ProRoot MTA showed equivalent performance in one study at six months follow-up [76] and in three studies at twelve months [17,76,77]. Although ProRoot MTA tended to present superior results in long-term follow-up, these differences were not statistically significant. The type of restorative treatment and the timing of permanent filling did not seem to influence the success of vital pulp treatment.

In permanent dentition, ProRoot MTA achieved better results in vital pulp treatment—pulpotomy and direct pulp capping—compared to other calcium silicate-based cements, except Biodentine, which had similar or better results at six months follow-up. In other comparisons, ProRoot MTA performed better than Portland, OrthoMTA, and RetroMTA at six- and twelve-months follow-up, and Endocem at twelve months. It showed similar performance to iRoot BP and TheraCal-LC at fifteen months and two months follow-up, respectively. This contrasts with the literature, which indicates that all calcium silicate-based materials, except resin-containing formulations, demonstrate favorable biological and histological responses nearly equivalent to ProRoot MTA [110,111]. Similar to primary dentition, there was significant diversity in clinical diagnosis (normal pulp or reversible pulpitis), treatment procedures (pulpotomy or direct pulp capping), restorative materials (composite, amalgam, stainless steel crown, ceramic crown, glass ionomer cement, or 3M ^TM^ Cavit ^TM^), and timing of restoration (immediate or deferred), which did not affect the clinical and radiographic results.

Generally, ProRoot MTA showed superior clinical success rates compared to most calcium silicate-based cements and was similar to Biodentine [5,96,97,98]. The differences were not sufficient to justify a different clinical performance, making it more relevant to consider material properties such as handling, setting time, biocompatibility, and tooth discoloration, as well as potential differences between commercial brands, to decide the best material for each patient (considering age and root tooth development) and specific treatment situation. Handling, setting time, and tooth discoloration are particularly important in direct pulp capping and should be considered when choosing a calcium silicate-based cement.

The present review, although limited by the number and quality of included studies for calcium silicate-based cements other than ProRoot MTA, has important strengths. Unlike previous systematic reviews, it aims to answer a specific and clinically relevant question regarding the choice of calcium silicate-based cements for each clinical situation using practical clinical outcome parameters. Although only histological analysis can evaluate the true condition of dental pulp tissue after vital pulp treatment, the follow-up periods for studies with histological evaluations (only two studies in primary dentition and seven in permanent dentition) were short-term, making it impossible to guarantee the long-term prognosis of calcium silicate-based cements in these treatments.

In daily clinical practice, there is no consistent association between clinical signs/symptoms (e.g., pulp sensibility testing, clinician’s assessment of patient pain history, direct observation of pulp tissue during and after hemostasis) and actual histopathology of diseased pulp [72,97]. Consequently, the diagnosis of reversible or irreversible pulpitis is not always clear, and the treatment decision between partial or full pulpotomy and root canal treatment is not always obvious. This study considered pulpotomy for both full and partial procedures.

In the present review, pulpotomy was also a treatment option for permanent dentition with a diagnosis of irreversible pulpitis in the studies [73,95,103]. This aligns with the American Association of Endodontists (AAE), which considers vital pulp therapy for symptomatic pulps [112]. Carious primary teeth diagnosed with a normal pulp requiring pulp therapy or with reversible pulpitis were treated with vital pulp procedures, as recommended in the American Academy of Pediatric Dentistry (AAPD) guidelines [21] and in agreement with most of the studies in this review. Irreversible pulpitis in primary teeth is usually treated with pulpectomy, which is considered more invasive, time-consuming, requiring more effort from children and experience from the dentist, non-biological, and results in a loss of the repair and regenerative properties characteristic of vital pulp therapy [65,113]. Recently, like in permanent dentition, the effectiveness of vital pulpotomy has been proven in the treatment of primary teeth affected by symptoms and signs of irreversible pulpitis. In a recent study, the success rate of Well-Root^TM^ PT Bioceramic putty (Vericom, Republic of Korea) was 100% clinically and radiologically after a one-year follow-up, while the success rate of MTA was 95% [93].

The review level of evidence can be classified as Level 1.a (systematic review of randomized controlled trials) according to Joanna Briggs Institute Levels of Evidence [114]. The review strengths are the assessment of randomized control trials only, and the review has a high level of evidence classification. While this systematic review provides valuable insights into the clinical, radiographic, and histological outcomes of calcium silicate-based cements in vital pulp therapy, several potential limitations of the included studies must be acknowledged. First, the risk of bias across studies varied, particularly in terms of randomization processes, blinding, and reporting outcomes, which may influence the reliability of the results. Additionally, there was considerable heterogeneity in the study populations, with differences in patient age, dental condition, and treatment protocols, which could affect the generalizability of the findings. The heterogeneity in interventions, such as the types of calcium silicate-based cements used and the variations in follow-up periods, further complicates direct comparisons across studies. Future research should focus on establishing standardized criteria for follow-up periods and success outcomes to enhance comparability across studies; it should also explore the long-term efficacy of different calcium silicate-based cements in diverse clinical scenarios, and evaluate if vital pulp therapy is also a treatment option for symptomatic pulps in primary teeth, as it is in permanent dentition.

## 5. Conclusions

In conclusion, the present comprehensive review evaluated the clinical, radiographic, and histological outcomes of various calcium silicate-based cements used in vital pulp therapy for both deciduous and permanent dentition across 40 randomized controlled trials. The findings revealed no significant short-term differences among different cements in primary teeth, with ProRoot MTA and Biodentine showing comparable success rates. However, ProRoot MTA tended to perform better in long-term outcomes for permanent teeth, except when compared with Biodentine, which demonstrated comparable or superior results at six months. This underscores the importance of selecting materials based on specific clinical scenarios and patient conditions. Despite the availability of numerous calcium silicate-based cements, ProRoot MTA and Biodentine remain the most reliable, highlighting the need for further high-quality, long-term studies to guide clinical decisions and establish optimal use for vital pulp therapy.

## Figures and Tables

**Table 1:** Combination of terms used for each electronic database.

Combination of Terms Used	Filters and Limits
PubMed	
#1 Search: **teeth AND pulpotomy AND mta AND biodentine** (((“teeth s”[All Fields] OR “teeths”[All Fields] OR “tooth”[MeSH Terms] OR “tooth”[All Fields] OR “teeth”[All Fields] OR “tooth s”[All Fields] OR “tooths”[All Fields]) AND (“pulpotomy”[MeSH Terms] OR “pulpotomy”[All Fields] OR “pulpotomies”[All Fields]) AND “mta”[All Fields] AND (“tricalcium silicate”[Supplementary Concept] OR “tricalcium silicate”[All Fields])) OR “biodentine”[All Fields])	Publication date: - From 1 January 1990–1 October, 2023 Species: - Human Article types: - Clinical trial - Controlled clinical trial - RCT
#2 Search: **teeth AND (dental pulp capping) AND MTA AND biodentine** ((“teeth s”[All Fields] OR “teeths”[All Fields] OR “tooth”[MeSH Terms] OR “tooth”[All Fields] OR “teeth”[All Fields] OR “tooth s”[All Fields] OR “tooths”[All Fields]) AND (“dental pulp capping”[MeSH Terms] OR (“dental”[All Fields] AND “pulp”[All Fields] AND “capping”[All Fields]) OR “dental pulp capping”[All Fields]) AND “mta”[All Fields] AND (“tricalcium silicate”[Supplementary Concept] OR “tricalcium silicate”[All Fields] OR “biodentine”[All Fields]))	
#3 Search: **teeth AND pulpotomy AND bioceramics** ((“teeth s”[All Fields] OR “teeths”[All Fields] OR “tooth”[MeSH Terms] OR “tooth”[All Fields] OR “teeth”[All Fields] OR “tooth s”[All Fields] OR “tooths”[All Fields]) AND (“pulpotomy”[MeSH Terms] OR “pulpotomy”[All Fields] OR “pulpotomies”[All Fields]) AND (“bioceramic”[All Fields] OR “bioceramics”[All Fields]))	
#4 Search: **teeth AND (dental pulp capping) AND MTA** ((“teeth s”[All Fields] OR “teeths”[All Fields] OR “tooth”[MeSH Terms] OR “tooth”[All Fields] OR “teeth”[All Fields] OR “tooth s”[All Fields] OR “tooths”[All Fields]) AND (“dental pulp capping”[MeSH Terms] OR (“dental”[All Fields] AND “pulp”[All Fields] AND “capping”[All Fields]) OR “dental pulp capping”[All Fields]) AND “MTA”[All Fields])	
#5 Search: **teeth AND (pulpotomy) AND MTA** ((“teeth s”[All Fields] OR “teeths”[All Fields] OR “tooth”[MeSH Terms] OR “tooth”[All Fields] OR “teeth”[All Fields] OR “tooth s”[All Fields] OR “tooths”[All Fields]) AND (“pulpotomy”[MeSH Terms] OR “pulpotomy”[All Fields] OR “pulpotomies”[All Fields]) AND “MTA”[All Fields])	
#6 Search: **teeth AND pulpotomy AND theracal** ((“teeth s”[All Fields] OR “teeths”[All Fields] OR “tooth”[MeSH Terms] OR “tooth”[All Fields] OR “teeth”[All Fields] OR “tooth s”[All Fields] OR “tooths”[All Fields]) AND (“pulpotomy”[MeSH Terms] OR “pulpotomy”[All Fields] OR “pulpotomies”[All Fields]) AND (“theracal”[Supplementary Concept] OR “theracal”[All Fields] OR “theracal”[All Fields])) #7 Search: **teeth AND (dental pulp capping) AND (calcium silicate cements)** ((“teeth s”[All Fields] OR “teeths”[All Fields] OR “tooth”[MeSH Terms] OR “tooth”[All Fields] OR “teeth”[All Fields] OR “tooth s”[All Fields] OR “tooths”[All Fields]) AND (“dental pulp capping”[MeSH Terms] OR (“dental”[All Fields] AND “pulp”[All Fields] AND “capping”[All Fields]) OR “dental pulp capping”[All Fields]) AND ((“calcium silicate”[Supplementary Concept] OR “calcium silicate”[All Fields]) AND (“cement s”[All Fields] OR “cementable”[All Fields] OR “cementation”[MeSH Terms] OR “cementation”[All Fields] OR “cementations”[All Fields] OR “cementing”[All Fields] OR “dental cementum”[MeSH Terms] OR (“dental”[All Fields] AND “cementum”[All Fields]) OR “dental cementum”[All Fields] OR “cement”[All Fields] OR “dental cements”[MeSH Terms] OR (“dental”[All Fields] AND “cements”[All Fields]) OR “dental cements”[All Fields] OR “cemented”[All Fields] OR “cements”[All Fields]))) #8 Search: **teeth AND (pulpotomy) AND (calcium silicate cements)** ((“teeth s”[All Fields] OR “teeths”[All Fields] OR “tooth”[MeSH Terms] OR “tooth”[All Fields] OR “teeth”[All Fields] OR “tooth s”[All Fields] OR “tooths”[All Fields]) AND (“pulpotomy”[MeSH Terms] OR “pulpotomy”[All Fields] OR “pulpotomies”[All Fields]) AND ((“calcium silicate”[Supplementary Concept] OR “calcium silicate”[All Fields]) AND (“cement s”[All Fields] OR “cementable”[All Fields] OR “cementation”[MeSH Terms] OR “cementation”[All Fields] OR “cementations”[All Fields] OR “cementing”[All Fields] OR “dental cementum”[MeSH Terms] OR (“dental”[All Fields] AND “cementum”[All Fields]) OR “dental cementum”[All Fields] OR “cement”[All Fields] OR “dental cements”[MeSH Terms] OR (“dental”[All Fields] AND “cements”[All Fields]) OR “dental cements”[All Fields] OR “cemented”[All Fields] OR “cements”[All Fields]))) #9 Search: **teeth AND (dental pulp capping) AND bioceramics** ((“teeth s”[All Fields] OR “teeths”[All Fields] OR “tooth”[MeSH Terms] OR “tooth”[All Fields] OR “teeth”[All Fields] OR “tooth s”[All Fields] OR “tooths”[All Fields]) AND (“dental pulp capping”[MeSH Terms] OR (“dental”[All Fields] AND “pulp”[All Fields] AND “capping”[All Fields]) OR “dental pulp capping”[All Fields]) AND (“bioceramic”[All Fields] OR “bioceramics”[All Fields])) #10 Search: **teeth AND Pulpotomy (bioactive cements)** ((“teeth s”[All Fields] OR “teeths”[All Fields] OR “tooth”[MeSH Terms] OR “tooth”[All Fields] OR “teeth”[All Fields] OR “tooth s”[All Fields] OR “tooths”[All Fields]) AND ((“pulpotomy”[MeSH Terms] OR “pulpotomy”[All Fields] OR “pulpotomies”[All Fields]) AND (“bioactivate”[All Fields] OR “bioactivated”[All Fields] OR “bioactivates”[All Fields] OR “bioactivating”[All Fields] OR “bioactivation”[All Fields] OR “bioactivations”[All Fields] OR “bioactive”[All Fields] OR “bioactives”[All Fields] OR “bioactivities”[All Fields] OR “bioactivity”[All Fields]) AND (“cement s”[All Fields] OR “cementable”[All Fields] OR “cementation”[MeSH Terms] OR “cementation”[All Fields] OR “cementations”[All Fields] OR “cementing”[All Fields] OR “dental cementum”[MeSH Terms] OR (“dental”[All Fields] AND “cementum”[All Fields]) OR “dental cementum”[All Fields] OR “cement”[All Fields] OR “dental cements”[MeSH Terms] OR (“dental”[All Fields] AND “cements”[All Fields]) OR “dental cements”[All Fields] OR “cemented”[All Fields] OR “cements”[All Fields]))) #11 Search: **teeth AND (dental pulp capping) AND (bioactive cements)** ((“teeth s”[All Fields] OR “teeths”[All Fields] OR “tooth”[MeSH Terms] OR “tooth”[All Fields] OR “teeth”[All Fields] OR “tooth s”[All Fields] OR “tooths”[All Fields]) AND (“dental pulp capping”[MeSH Terms] OR (“dental”[All Fields] AND “pulp”[All Fields] AND “capping”[All Fields]) OR “dental pulp capping”[All Fields]) AND ((“bioactivate”[All Fields] OR “bioactivated”[All Fields] OR “bioactivates”[All Fields] OR “bioactivating”[All Fields] OR “bioactivation”[All Fields] OR “bioactivations”[All Fields] OR “bioactive”[All Fields] OR “bioactives”[All Fields] OR “bioactivities”[All Fields] OR “bioactivity”[All Fields]) AND (“cement s”[All Fields] OR “cementable”[All Fields] OR “cementation”[MeSH Terms] OR “cementation”[All Fields] OR “cementations”[All Fields] OR “cementing”[All Fields] OR “dental cementum”[MeSH Terms] OR (“dental”[All Fields] AND “cementum”[All Fields]) OR “dental cementum”[All Fields] OR “cement”[All Fields] OR “dental cements”[MeSH Terms] OR (“dental”[All Fields] AND “cements”[All Fields]) OR “dental cements”[All Fields] OR “cemented”[All Fields] OR “cements”[All Fields]))) #12 Search: **teeth AND (dental pulp capping) AND (calcium-enriched mixture cement)** ((“teeth s”[All Fields] OR “teeths”[All Fields] OR “tooth”[MeSH Terms] OR “tooth”[All Fields] OR “teeth”[All Fields] OR “tooth s”[All Fields] OR “tooths”[All Fields]) AND (“dental pulp capping”[MeSH Terms] OR (“dental”[All Fields] AND “pulp”[All Fields] AND “capping”[All Fields]) OR “dental pulp capping”[All Fields]) AND (“calcium enriched mixture cement”[Supplementary Concept] OR “calcium enriched mixture cement”[All Fields] OR “calcium enriched mixture cement”[All Fields])) #13 Search: **teeth AND Pulpotomy AND (calcium-enriched mixture cement)** ((“teeth s”[All Fields] OR “teeths”[All Fields] OR “tooth”[MeSH Terms] OR “tooth”[All Fields] OR “teeth”[All Fields] OR “tooth s”[All Fields] OR “tooths”[All Fields]) AND (“pulpotomy”[MeSH Terms] OR “pulpotomy”[All Fields] OR “pulpotomies”[All Fields]) AND (“calcium enriched mixture cement”[Supplementary Concept] OR “calcium enriched mixture cement”[All Fields] OR “calcium enriched mixture cement”[All Fields])) #14 Search: **teeth AND Pulpotomy AND (Portland cement)** ((“teeth s”[All Fields] OR “teeths”[All Fields] OR “tooth”[MeSH Terms] OR “tooth”[All Fields] OR “teeth”[All Fields] OR “tooth s”[All Fields] OR “tooths”[All Fields]) AND (“pulpotomy”[MeSH Terms] OR “pulpotomy”[All Fields] OR “pulpotomies”[All Fields]) AND (“Portland”[All Fields] AND (“cement s”[All Fields] OR “cementable”[All Fields] OR “cementation”[MeSH Terms] OR “cementation”[All Fields] OR “cementations”[All Fields] OR “cementing”[All Fields] OR “dental cementum”[MeSH Terms] OR (“dental”[All Fields] AND “cementum”[All Fields]) OR “dental cementum”[All Fields] OR “cement”[All Fields] OR “dental cements”[MeSH Terms] OR (“dental”[All Fields] AND “cements”[All Fields]) OR “dental cements”[All Fields] OR “cemented”[All Fields] OR “cements”[All Fields]))) #15 Search: **teeth AND (dental pulp capping) AND (Portland cement)** ((“teeth s”[All Fields] OR “teeths”[All Fields] OR “tooth”[MeSH Terms] OR “tooth”[All Fields] OR “teeth”[All Fields] OR “tooth s”[All Fields] OR “tooths”[All Fields]) AND (“dental pulp capping”[MeSH Terms] OR (“dental”[All Fields] AND “pulp”[All Fields] AND “capping”[All Fields]) OR “dental pulp capping”[All Fields]) AND (“Portland”[All Fields] AND (“cement s”[All Fields] OR “cementable”[All Fields] OR “cementation”[MeSH Terms] OR “cementation”[All Fields] OR “cementations”[All Fields] OR “cementing”[All Fields] OR “dental cementum”[MeSH Terms] OR (“dental”[All Fields] AND “cementum”[All Fields]) OR “dental cementum”[All Fields] OR “cement”[All Fields] OR “dental cements”[MeSH Terms] OR (“dental”[All Fields] AND “cements”[All Fields]) OR “dental cements”[All Fields] OR “cemented”[All Fields] OR “cements”[All Fields]))) #16 Search: **teeth AND (pulpotomy) AND PulpGuard** (“teeth”[All Fields] AND “pulpotomy”[All Fields]) AND “PulpGuard”[All Fields] #17 Search: **teeth AND (dental pulp capping) AND PulpGuard** (“teeth”[All Fields] AND (“dental”[All Fields] AND “pulp”[All Fields] AND “capping”[All Fields]) AND “PulpGuard”[All Fields]) #18 Search: **teeth AND (dental pulp capping)** ((“teeth s”[All Fields] OR “teeths”[All Fields] OR “tooth”[MeSH Terms] OR “tooth”[All Fields] OR “teeth”[All Fields] OR “tooth s”[All Fields] OR “tooths”[All Fields]) AND (“dental pulp capping”[MeSH Terms] OR (“dental”[All Fields] AND “pulp”[All Fields] AND “capping”[All Fields]) OR “dental pulp capping”[All Fields])) #19 Search: **teeth AND pulpotomy** ((“teeth s”[All Fields] OR “teeths”[All Fields] OR “tooth”[MeSH Terms] OR “tooth”[All Fields] OR “teeth”[All Fields] OR “tooth s”[All Fields] OR “tooths”[All Fields]) AND (“pulpotomy”[MeSH Terms] OR “pulpotomy”[All Fields] OR “pulpotomies”[All Fields])) #20 Search: **teeth AND (vital pulp therapy) AND mta** ((“teeth s”[All Fields] OR “teeths”[All Fields] OR “tooth”[MeSH Terms] OR “tooth”[All Fields] OR “teeth”[All Fields] OR “tooth s”[All Fields] OR “tooths”[All Fields]) AND ((“vital signs”[MeSH Terms] OR (“vital”[All Fields] AND “signs”[All Fields]) OR “vital signs”[All Fields] OR “vital”[All Fields] OR “vitally”[All Fields] OR “vitals”[All Fields]) AND (“dental pulp”[MeSH Terms] OR (“dental”[All Fields] AND “pulp”[All Fields]) OR “dental pulp”[All Fields] OR “pulp”[All Fields]) AND (“therapeutics”[MeSH Terms] OR “therapeutics”[All Fields] OR “therapies”[All Fields] OR “therapy”[MeSH Subheading] OR “therapy”[All Fields] OR “therapy s”[All Fields] OR “therapys”[All Fields])) AND “mta”[All Fields])	
Lilacs	
#1 Search: (tw:(dente)) OR (tw:(diente)) AND (tw:(MTA)) AND (tw:(proteção pulpar) OR (tw:(protecion pulpar))	Article types:
#2 Search: (tw:(diente)) OR (tw:(dente)) AND (tw:(pulpotomia)) AND (tw:(MTA))	- Controlled clinical trial
Cochrane Collaboration	
#1 Search: (MTA)	N/F

N/F: no filter.

**Table 2:** Eligibility criteria.

Code	Limit/Criteria	Description
Inclusion criteria		
I-1	Criteria	Clinical trials that compared at least two calcium silicate-based cements
I-2	Limit	Human studies
I-3	Criteria	Randomized controlled trials (RCTs)
I-4	Limit	Vital pulp treatments only
I-5	Limit	Each tooth was evaluated as a whole
I-6	Criteria	Sample size was given (number of teeth)
I-7	Criteria	Clearly states the pulp tissue health for at least one time period, by clinical and radiographical or histological evaluation
I-8	Criteria	The studies have to specify commercial materials (brands) used
I-9	Criteria	The studies should specify at least one strict follow-up period
I-CASP	Criteria	CASP–RCT critical appraisal was equal or greater than 50%
Exclusion criteria		
E-1	Criteria	Root resorptions, pulpectomies, and apical barriers
E-2	Limit	Shorter follow-up study using the same sample of another included study presenting a longer follow-up
E-3	Limit	Sample was partially analyzed in another included study
E-4	Limit	Indirect pulp capping treatments

**Table 3:** List of the studies excluded from the review.

Study	Inclusion Factor Absent (Code) *	Excluded Data	Reason
Abuelniel et al. (2021) [43]	I-7	All	This study did not clearly state the pulp tissue health for at least one time period, only the root development.
Abuelniel et al. (2020) [44]	I-7	All	This study did not clearly state the pulp tissue health for at least one time period, only the root development.
Asgary et al. (2013) [45]	E-2	All	Short follow-up study using the same sample of another included study presenting a longer follow-up (Asgary et al. (2017) [73]).
Bokhari et al. (2016) [46]	I-7	All	This study did not clearly state the pulp tissue health for at least one time period, only the response to pain.
Cardoso-Silva et al. (2011) [47]	I-3, I-5	All	The results were presented by the number of the roots, not by the number of the teeth.
Carti and Oznurhan (2017) [48]	I-8	All	The MTA commercial company was not specified.
Cuadros-Fernández et al. (2016) [49]	I-8	All	The MTA commercial company was not specified. The results present the overall success.
Eghbal et al. (2009) [50]	I-1	All	Only one calcium silicates cement was used.
Fouad andYoussef (2013) [51]	I-3	All	The study was not randomized.
Ghajari et al. (2010) [52]	E-2	All	Short follow-up study using the same sample of another included study presenting a longer follow-up (Ghajari et al. (2013) [74]).
Guang et al. (2022) [53]	I-7	All	The clinical and radiographical follow-up criteria were not indicated.
Hegde et al. (2017) [54]	I-3	All	The study was not randomized.
Juneja and Kulkarni (2017) [55]	I-8	All	The MTA commercial company was not specified.
Linu et al. (2017) [56]	I-3	All	This is a retrospective study.
Liu et al. (2015) [57]	I-2	All	This study used cellular lines and animal models.
Meligy et al. (2016) [58]	I-1	All	Only one calcium silicates cement was used.
Mythraiye et al. (2019) [59]	I-8	All	The studies have to specify commercial materials used.
Niranjani et al. (2015) [60]	I-CASP	All	Under 50% score at CASP–RCT.
Nosrat et al. (2013) [61]	I-5	All	The results were presented by the number of the roots, not by the number of the teeth.
Nosrat et al. (2013) [62]	I-5, I-CASP	All	Under 50% score at CASP–RCT. The results were presented by the number of the roots, not by the number of the teeth.
Nowicka et al. (2013) [63]	I-3	All	The study is not randomized. Disparity between the number of teeth for experimental group (11 teeth) and control group (6 teeth).
Parinyaprom et al. (2018) [64]	I-9	All	The studies should specify at least one follow-up period.
Petrou et al. (2014) [65]	E-4	All	Indirect pulp capping treatments were excluded.
Song et al. (2015) [66]	E-2	All	Short follow-up study using the same sample of another included study presenting a longer follow-up.
Swarup et al. (2014) [67]	I-1	All	Only one calcium silicates cement was used.
Tan et al. (2020) [68]	I-1	All	Only one calcium silicates cement was used.
Togaru et al. (2016) [69]	I-CASP	All	Under 50% score at CASP–RCT.
Tzanetakis et al. (2023) [70]	I-9	All	Clinical and radiographic follow-up evaluation was performed for a median time of 2 years and did not specify the follow-up periods.
Uesrichai et al. (2019) [71]	I-9	All	The studies should specify at least one follow-up period.
Zarrabi et al. (2011) [72]	E-2	All	The same sample of another included study (Zarrabi et al. (2010) [75]), and this study is regarding immunohistochemical test.

* Codes for inclusion factors are listed in Table 2.

**Table 4:** Overall agreement scores for the CASP–Randomized Controlled Trial questionnaire.

#	Critical Appraisal Skills Programme (CASP) Question	Overall Agreement between Evaluators
1	Did the trial address a clearly focused issue?	97.5%
2	Was the assignment of patients to treatments randomized?	100%
3	Were all of the patients who entered the trial properly accounted for at its conclusion?	100%
4	Were patients, health workers and study personnel ‘blind’ to treatment?	85.0%
5	Were the groups similar at the start of the trial?	80.0%
6	Aside from the experimental intervention, were the groups treated equally?	100%
7	How large was the treatment effect?	80.0%
8	How precise was the estimate of the treatment effect?	87.5%
9	Can the results be applied to the local population, or in your context?	52.5%
10	Were all clinically important outcomes considered?	82.5%
11	Are the benefits worth the harms and costs?	97.5%

**Table 5:** Success criteria in deciduous teeth studies regarding the clinical and radiographic parameters.

Study	Clinical Criteria *	Radiographic Criteria *
MTA vs. Biodentine		
Vilella-Pastor et al. (2021) [76]	The clinical success criteria were: absence of symptoms of pain, absence of abscess, fistula or swelling, and absence of pathological mobility.	The radiographic success criteria were: absence of periapical radiolucency or interradicular furcation, absence of internal or external root resorption, and absence of widening of the periodontal ligament.
Çelik et al. (2019) [17]	The absence of spontaneous pain, pathologic mobility, tenderness to percussion, swelling, fistula, and gingival inflammation was considered as a clinical success.	Absence of internal/external root resorption and periapical/furcal radiolucency was considered as a radiographic success. Calcific metamorphosis of the pulp was not considered a failure.
Bani et al. (2017) [16]	The criteria for clinical success were: the absence of tenderness to percussion; swelling; fistulation; spontaneous pain; or pathologic mobility.	The criteria for radiographic success were: the absence of postoperative radiographic pathology, such as external or internal root resorption; furcal or periapical radiolucency; widened periodontal ligament spaces.
Guven et al. (2017) ^1^ [77]	The pulpotomized tooth was considered to be a clinical success if no swelling, pain, fistula, or pathologic mobility occurred.	Teeth were considered to be a radiographic success if they showed no evidence of internal or external resorption or periradicular radiolucency. Pulp canal obliteration (PCO) was not regarded as a failure.
Rajasekharan et al. (2017) [78]	This scoring system was devised to represent severity of changes but not to label an individual tooth as “success” or “failure”: 1—Asymptomatic/6-month recall: pathology: absent; normal functioning; naturally exfoliated; exfoliation prematurely due to ectopic eruption. 2—Slight discomfort/short-lived/3-month recall: pathology questionable; percussion sensitivity; chewing sensitivity, short-lasting; gingival inflammation (due to poor oral hygiene); mobility (physiological) >1 mm but <2 mm. 3—Minor discomfort/short-lived/1-month recall: pathology initial changes present; chewing sensitivity, long-lasting; gingival swelling (not due to poor oral hygiene); periodontal pocket formation (no exudate); mobility >2 mm but <3 mm. 4—Major discomfort/long-lived/extract immediately; pathology late changes present; spontaneous pain; gingival swelling (not due to poor oral hygiene); periodontal pocket formation (exudate); sinus tract present; mobility ≥3 mm; premature tooth loss due to pathology.	This scoring system was devised to represent severity of changes but not to label an individual tooth as “success” or “failure”: 1—No changes present/6-month follow-up: internal root canal form tapering from chamber to the apex; PDL/periapical regions; normal width and trabeculation. 2—External changes are not allowed (widened periodontal ligament widening—PDL); abnormal inter-radicular trabeculation or variation in radiodensity; internal resorption acceptable (nonperforated); calcific metamorphosis is acceptable and defined as: uniformly thin root canal; shape (no tapering); variation in radiodensity from canal to canal (one cloudier than the other); dentine bridge formation (one or more canals). 3—Pathological changes present/1-month follow-up: external changes are present, but not large; mildly widened PDL; minor inter-radicular radiolucency with trabeculation still present; minor external root resorption; internal resorption changes are acceptable, but not if external change is also present (perforated form). 4—Pathological changes present/extract immediately: frank osseous radiolucency present, endangering permanent successor.
Kusum et al. (2015) [79]	Clinical success—Pathology: absent/questionable; normal functioning; mobility (physiological) ≤2 mm; percussion sensitivity; gingival inflammation (due to poor oral hygiene).	Radiographic success—Internal root canal form tapering from chamber to the apex; periodontal ligament (PDL)/periapical regions; normal width and trabeculation; external changes are not allowed; (widened PDL) widening, abnormal inter-radicular trabeculation or variation in radiodensity pathological; internal resorption acceptable (not perforated); calcific metamorphosis is acceptable and defined as: uniformly thin root canal; shape (non-tapering); variation in radiodensity from canal to canal (one cloudier than the other).
ProRoot MTA vs. CEM		
Ghajari et al. (2013) [74]	Symptoms such as pain, swelling, tenderness to pressure, and signs such as presence of sinus tract, swelling and tenderness to percussion were evaluated as the clinical criteria for failure.	Internal and/or external root resorption, interradicular radiolucency, and periapical lesions were assessed as the radiographic criteria for failure.
Malekafzali et al. (2011) [80]	The treatment outcome was classified as a failure when one or more of the following signs were present: swelling/abscess, sinus tract, spontaneous pain, and/or pathological mobility.	The treatment outcome was classified as a failure when one or more of the following signs were present: radiograph evaluation detects signs of furcation radiolucency, periapical bone destruction, internal root resorption, and/or pathological external root resorption.
ProRoot MTA vs. MTA Plus		
Guven et al. (2017) ^1^ [77]	The pulpotomized tooth was considered to be clinical success if no swelling, pain, fistula, or pathologic mobility occurred.	Teeth were considered to be a radiographic success if they showed no evidence of internal or external resorption or periradicular radiolucency. Pulp canal obliteration (PCO) was not regarded as a failure.
ProRoot MTA vs. NeoMTA Plus		
Alsanouni and Bawazir (2019) [81]	At each follow-up appointment, the treatment was considered to have clinical failure if one of the following signs and symptoms was present: spontaneous pain; tenderness to percussion or palpation; soft tissue swelling; sinus tract or fistula; or pathologic tooth mobility.	The treatment was considered to have radiographic failure if one of the following signs were present: widening of the periodontal ligament space; furcal or periapical radiolucency; or pathological external or internal root resorption.
ProRoot MTA vs. Angelus MTA		
Celik et al. (2013) [20]	The following criteria were used for the determination of clinical success: absence of spontaneous pain and/or sensitivity to palpation/percussion; absence of fistula, swelling, and/or abnormal mobility.	The following criteria were used for the determination of radiographic success: absence of radiolucency at the inter-radicular and/or periapical regions, as determined by conventional periapical radiographs taken at all control appointments; absence of pulp canal obliteration (fully obliterated canals); and absence of internal or external (pathologic) resorption that was not compatible with a normal exfoliation process.
White MTA ProRoot vs. Gray MTA ProRoot		
Agami et al. (2004) [82]	Teeth were scored as clinical successes if they had no evidence of: pain symptoms; tenderness to percussion; swelling; fistulation; or pathologic mobility.	Teeth were scored as radiographic successes if they showed no evidence of: radicular radiolucency; internal or external root resorption; periodontal ligament space widening.
ProRoot MTA vs. TheraCal-LC		
Hassanpour et al. (2023) [83]	Presenting either of sinus tract, swelling, periapical lesion, spontaneous pain or long-lasting pain, tenderness to palpation and percussion, internal/external root resorption, or interradicular radiolucency was accounted as the treatment failure.	
Erfanparast et al. (2018) [84]	The presence of one of the following signs or symptoms was considered as failure of treatment: pain, swelling, sinus tract, pathologic mobility, tenderness to palpation, sensitivity to percussion.	The presence of one of the following signs or symptoms was considered as failure of treatment: radiographic sign of internal and/or external root resorption, periodontal space widening, inter-radicular radiolucency, periapical lesions, and recurrent caries under the restoration.
ProRoot MTA vs. OrthoMTA		
Kang et al. (2015) ^2^ [85]	Clinical failure: spontaneous pain and/or sensitivity to palpation/percussion; fistula, gingival redness, and swelling and/or mobility.	Radiographic failure: bone resorption at the periapical and/or interradicular regions; periodontal ligament (PDL) space widening; and external/internal root resorption that were not related to a normal exfoliation process.
ProRoot MTA vs. RetroMTA		
Kang et al. (2015) ^2^ [85]	Clinical failure: spontaneous pain and/or sensitivity to palpation/percussion; fistula, gingival redness, and swelling and/or mobility.	Radiographic failure: bone resorption at the periapical and/or interradicular regions; PDL space widening; and external/internal root resorption that was not related to a normal exfoliation process.
ProRoot MTA vs. Endocem		
Joo et al. (2023) [86]	The clinical success criteria were (1) the absence of pathologic mobility; (2) the absence of spontaneous pain and/or sensitivity to palpation/percussion; and (3) the absence of gingival swelling or fistula.	The radiographic success criteria were (1) the absence of internal/external root resorption; and (2) the absence of periapical/furcal radiolucency or bone resorption. If any clinical or radiological failure occurred, it was considered a failure.
ProRoot MTA vs. BC Putty		
Joo et al. (2023) [86]	The clinical success criteria were (1) the absence of pathologic mobility; (2) the absence of spontaneous pain and/or sensitivity to palpation/percussion; and (3) the absence of gingival swelling or fistula.	The radiographic success criteria were (1) the absence of internal/external root resorption; and (2) the absence of periapical/furcal radiolucency or bone resorption. If any clinical or radiological failure occurred, it was considered a failure.
ProRoot MTA vs. Portland		
Yildirim et al. (2016) [87]	The teeth were evaluated as successful or unsuccessful according to the above criteria. Spontaneous pain, swelling, fistula were indications for tooth removal.	The teeth were evaluated as successful or unsuccessful according to the above criteria. Radiolucency of the periapical or furcation, and pathological external root resorption were indications for tooth removal. Teeth with radiographic pulp canal obliteration and internal root resorption, but with no clinical symptoms, were monitored but not removed.
OrthoMTA vs. RetroMTA		
Kang et al. (2015) ^2^ [85]	Clinical failure: spontaneous pain and/or sensitivity to palpation/percussion; fistula, gingival redness, and swelling and/or mobility.	Radiographic failure: bone resorption at the periapical and/or interradicular regions; PDL space widening; and external/internal root resorption that was not related to a normal exfoliation process.
MTA Plus vs. Biodentine		
Guven et al. (2017) ^1^ [77]	The pulpotomized tooth was clinical success if no swelling, pain, fistula, or pathologic mobility occurred.	Teeth were a radiographic success if they showed no evidence of internal or external resorption or periradicular radiolucency. Pulp canal obliteration (PCO) was not regarded as a failure.
NeoMTA vs. NeoPUTTY		
Alqahtani et al. (2023) [88]	At each follow-up, the treatment was considered a clinical failure if one or more of the following signs and symptoms were present: pain; swelling; pathological mobility; sinus tract; and tenderness to percussion.	The treatment was considered a radiographic failure if one or more of the following signs were present: widening of the PDL; internal or ex- ternal root resorption; and furcal and/or periapical radiolucency.
Angelus MTA vs. Portland		
Oliveira et al. (2013) [89]	Clinical success was confirmed: no spontaneous pain, mobility, swelling and fistula.	Radiographic success: internal root resorption and furcation radiolucency were absent. Dentine bridge formation and intra-canal calcifications were not considered as failures.
Sakai et al. (2009) [90]	Clinical success: teeth with no spontaneous pain, mobility, swelling, fistula, or smell.	Radiographic success: internal root resorption and furcation radiolucency were absent. Dentine bridge formation was also considered a radiographic success, and intracanal calcifications were not considered as failures.
Angelus MTA vs. Bio-C Pulpo		
Lima et al. (2020) [91]	The treatment was considered a clinical failure if one of the following signs or symptoms was detected: spontaneous pain, tenderness to percussion or palpation, pathologic mobility, swelling, fistula, or gingival inflammation.	The treatment was considered a radiographic failure if one of the following signs were detected: pathologic external root resorption, or no, self-limited or stable internal root resorption, or else periapical/furcal radiolucency.
Angelus MTA vs. Biodentine		
Ramanandvignesh et al. (2020) [92]	All teeth were evaluated clinically and radiographically based on AAPD criteria: (1) absence of spontaneous pain and/or sensitivity to pressure; (2) absence of sinus, fistula, edema, and/ or abnormal mobility; (3) absence of radiolucency at the interradicular and/or periapical regions; (4) absence of internal or external root resorption.	
Angelus MTA vs. BC putty		
Alnassar et al. (2022) [93]	The treatment was considered clinically successful in the absence of pain, swelling, fistula, and pain on percussion and bites.	Treatment was considered successful radiologically in the absence of periodontal ligament widening, and internal or external root resorption, in addition to evaluating the presence of radiolucency in the furcation area according to the following scores: Score 0: no radiolucency; score 1: radiolucency between 1⁄4 of furcation to periapical areas; score 2: radiolucency between 1⁄4 and 1⁄2 of furcation to periapical areas; and score 3: radiolucency more than 1⁄2 of furcation to periapical areas. The treated teeth with a score of 1 or 2 were considered successful according to the previous criteria.

* The articles were directly cited; ^1^ References referring to the same study; ^2^ References referring to the same study.

**Table 6:** Success criteria in permanent teeth studies regarding the clinical and radiographic parameters.

Study	Clinical Criteria *	Radiographic Criteria *
ProRoot MTA vs. Biodentine		
Singh et al. (2023) [94]	Teeth with no clinical signs and symptoms (pain, tenderness to percussion, sinus tract/swelling	No evidence of pathosis such as root resorption, furcal, or periapical rarefaction on the recall radiographs were categorized as clinically/radiographically successful, respectively.
Taha et al. (2022) [95]	Absence of clinical signs and symptoms indicative of pulpal pathosis (pain, tenderness to percussion).	Absence of pathosis on recall radiograph, i.e., root resorption, new furcal or periapical lesion. Complete radiographic healing or reduction in the size of periapical rarefaction if it was present preoperatively.
Uyar and Alaçam (2021) [96]	In clinical evaluation, percussion tenderness of teeth and palpation of soft tissue around the teeth, the formation of abscess and fistula were examined. Postoperative pain and type and duration of the pain were recorded. If postoperative pain or any clinical symptom was detected, it was considered as clinical failure.	Periapical radiographs were obtained preoperatively and postoperatively to assess the condition of periradicular tissues with Image Plate System (Digora^®^, Soredex, Helsinki, Finland). But, if clinical symptoms accompanied with the one of the radiographic failures, it was considered as failure and treated with apexification. However, if radiographic failure was seen without any clinical symptoms, it was not treated with apexification, continue to follow-up until the observation of any clinical signs or symptoms.
Bakhtiar et al. (2017) ^1^ [97]	Clinical tests and electric pulp test were performed to assess pulp vitality.	Radiographs to determine any signs of periapical pathology.
Brizuela et al. (2017) [98]	Clinical success was defined as a tooth with no pain, normal sensitivity tests, no facial edema, and no fistula.	No internal or external resorption, no periradicular disease, periodontal ligaments of normal width.
Nowicka et al. (2015) [99]	Electric pulp testing.	Radiographic evaluation.
ProRoot MTA vs. Angelus MTA		
Katge and Patil (2017) [100]	The treatment was considered to be clinically successful when the tooth remained asymptomatic and vital with a standard response to electrical pulp vitality test.	The treatment was considered to be radiographically successful when a dentin bridge was present over the lesion with the absence of periapical radiolucency and no periodontal ligament space widening, calcification, and internal and external resorption.
ProRoot MTA vs. iRoot BP		
Azimi et al. (2014) [102]	Presence or absence of postoperative sensitivity was observed to evaluate the periapical status of the teeth by the main operator.	Periapical radiograph of the teeth was taken to evaluate the periapical status of the teeth by the main operator.
ProRoot MTA vs. CEM		
Asgary et al. (2022) [103]	The outcome of clinical success/failure was determined by the subjective symptoms and objective observation of inflammation/infection. Objective signs, e.g., abscess, swelling, sinus tract, redness, pain, and tenderness to percussion.	The outcome of radiographic success was classified using a modification of Strindberg’s criteria: teeth with normal contour/width of periodontal ligament (PDL) were deliberated as success, and teeth with periapical radiolucency were reported as failure.
Asgary et al. (2017) [73]	Clinical failure was determined by: subjective reports of symptoms by subject. Objective signs included abscess, swelling, sinus tract infection, redness and tenderness associated with tooth.	The outcome of radiographic success: teeth with normal contour and width of PDL were judged as healed, teeth with a decreased size of the periapical radiolucency were judged as healing. Teeth unchanged, increased in size of the periapical radiolucency, or with the appearance of new periapical radiolucency were judged as failed. Internal/external root resorption and pulp obliteration were also assessed radiographically.
ProRoot MTA vs. OrthoMTA		
Kang et al. (2017) ^2^ [105]	Spontaneous pain (Visual Analogue Scale ≥1, symptomatic) and/or sensitivity to palpation/percussion; periodontal conditions (gingival redness and swelling).	Periapical radiolucency; pathological root resorption.
ProRoot MTA vs. Retro MTA		
Kang et al. (2017) ^2^ [105]	Spontaneous pain (Visual Analogue Scale ≥ 1, symptomatic) and/or sensitivity to palpation/percussion; periodontal conditions (gingival redness and swelling).	Periapical radiolucency; pathological root resorption.
ProRoot MTA vs. TotalFill		
Taha et al. (2022) [95]	Absence of clinical signs and symptoms indicative of pulpal pathosis (pain, tenderness to percussion).	Absence of pathosis on recall radiograph, i.e., root resorption, new furcal or periapical lesion. Complete radiographic healing or reduction in the size of periapical rarefaction if it was present preoperatively.
ProRoot MTA vs. Endocem MTA		
Jang et al. (2015) [107]	Treatment success was defined by cases in which the tooth exhibited a positive response to the pulp sensibility test without any evidence of irreversible pulpitis or pulp necrosis in the clinical examination. The following results were taken to indicate treatment failure: a negative response to the pulp sensibility test, spontaneous pain that was not resolved with analgesics.	The following results were taken to indicate treatment failure: well-defined apical radiolucency on the periapical radiograph.
ProRoot MTA vs. TheraCal-LC		
Bakhtiar et al. (2017) ^1^ [97]	Clinical tests and electric pulp test were performed to assess pulp vitality.	Radiographs to determine any signs of periapical pathology.
Angelus MTA vs. Biodentine		
Awawdeh et al. (2018) [108]	Clinical examinations were performed to detect soft tissue swelling, the integrity of the coronal restoration, crown discoloration. Tooth vitality was judged by a positive response to cold tests using Endo-Ice F (Coltene/Whaledent, Langenau, Germany). Treatment was considered successful based on the following features: absence of signs and symptoms of pulpal pathosis; lack of pain and tenderness to percussion; no soft tissue swelling, fistula, or abnormal mobility.	Radiographic examinations were performed to detect periapical status, the formation of a dentin bridge, pulpal calcifications, and canal obliteration. Treatment was considered successful based on the following features: absence of signs and of pulpal pathosis; absence of periapical rarefaction, internal or external resorption, and root canal obliteration; and normal pulp viability.
OrthoMTA vs. Retro MTA		
Kang et al. (2017) ^2^ [105]	Spontaneous pain (Visual Analogue Scale ≥1, symptomatic) and/or sensitivity to palpation/percussion; periodontal conditions (gingival redness and swelling).	Periapical radiolucency; pathological root resorption.
Angelus MTA vs. CEM		
MTA+ vs. Biodentine		
Peskersoy et al. (2021) [109]	Clinical Scoring Criteria: 1 (Asymptomatic)—Pathology: Absent; Functioning: Normal; Percussion and Sensitivity: Asymptomatic; Mobility: (0). 2 (Slight Discomfort)—Pathology: Questionable; Functioning: Chewing sensitivity, short-lasting; Percussion and Sensitivity: (-) and only on cold; Mobility: (Grade I). 3 (Minor Discomfort)—Pathology: Initial changes present; Functioning: Chewing sensitivity, long-lasting; Percussion and Sensitivity: (+) and only on cold; Mobility: (Grade I or II). 4 (Major Discomfort)—Pathology: Late changes present; Functioning: Spontaneous pain Percussion and Sensitivity: (+) and on cold and hot; Mobility: (Grade II or III).	Radiographic Scoring Criteria: 1 (No changes present)—PDL: Normal Width; Periapical Region: Normal; Root and Alveolar Bone Status: Normal; Complete dentine bridge formation (>1 mm thickness). 2 (Questionable pathological changes present)—PDL: Slightly Widened PDL; Periapical Region: Normal; Root and Alveolar Bone Status: Abnormal; Partial dentine bridge formation (0.5 e1 mm thickness). 3 (Minor Pathological changes present)—PDL: Widened PDL; Periapical Region: Minor external root resorption; Root and Alveolar Bone Status: External changes; Initial dentine bridge formation (<0.5 mm thickness). 4 (Major Pathological changes present)—PDL: Widened PDL; Periapical Region: Radiolucency present; Root and Alveolar Bone Status: Radiolucency present (No dentin bridge formation).
MTA+ vs. TheraCal-LC		
Peskersoy et al. (2021) [109]	Clinical Scoring Criteria: 1 (Asymptomatic)—Pathology: Absent; Functioning: Normal; Percussion and Sensitivity: Asymptomatic; Mobility: (0). 2 (Slight Discomfort)—Pathology: Questionable; Functioning: Chewing sensitivity, short-lasting; Percussion and Sensitivity: (-) and only on cold; Mobility: (Grade I). 3 (Minor Discomfort)—Pathology: Initial changes present; Functioning: Chewing sensitivity, long-lasting; Percussion and Sensitivity: (+) and only on cold; Mobility: (Grade I or II). 4 (Major Discomfort)—Pathology: Late changes present; Functioning: Spontaneous pain Percussion and Sensitivity: (+) and on cold and hot; Mobility: (Grade II or III).	Radiographic Scoring Criteria: 1 (No changes present)—PDL: Normal Width; Periapical Region: Normal; Root and Alveolar Bone Status: Normal; Complete dentine bridge formation (>1 mm thickness). 2 (Questionable pathological changes present)—PDL: Slightly Widened PDL; Periapical Region: Normal; Root and Alveolar Bone Status: Abnormal; Partial dentine bridge formation (0.5 e1 mm thickness). 3 (Minor Pathological changes present)—PDL: Widened PDL; Periapical Region: Minor external root resorption; Root and Alveolar Bone Status: External changes; Initial dentine bridge formation (<0.5 mm thickness). 4 (Major Pathological changes present)—PDL: Widened PDL; Periapical Region: Radiolucency present; Root and Alveolar Bone Status: Radiolucency present (No dentin bridge formation).
Biodentine vs. TotalFill		
Taha et al. (2022) [95]	Absence of clinical signs and symptoms indicative of pulpal pathosis (pain, tenderness to percussion).	Absence of pathosis on recall radiograph, i.e., root resorption, new furcal or periapical lesion. Complete radiographic healing or reduction in the size of periapical rarefaction if it was present preoperatively.
Biodentine vs. TheraCal-LC		
Peskersoy et al. (2021) [109]	Clinical Scoring Criteria: 1 (Asymptomatic)—Pathology: Absent; Functioning: Normal; Percussion and Sensitivity: Asymptomatic; Mobility: (0). 2 (Slight Discomfort)—Pathology: Questionable; Functioning: Chewing sensitivity, short-lasting; Percussion and Sensitivity: (-) and only on cold; Mobility: (Grade I). 3 (Minor Discomfort)—Pathology: Initial changes present; Functioning: Chewing sensitivity, long-lasting; Percussion and Sensitivity: (+) and only on cold; Mobility: (Grade I or II). 4 (Major Discomfort)—Pathology: Late changes present; Functioning: Spontaneous pain Percussion and Sensitivity: (+) and on cold and hot; Mobility: (Grade II or III).	Radiographic Scoring Criteria: 1 (No changes present)—PDL: Normal Width; Periapical Region: Normal; Root and Alveolar Bone Status: Normal; Complete dentine bridge formation (>1 mm thickness). 2 (Questionable pathological changes present)—PDL: Slightly Widened PDL; Periapical Region: Normal; Root and Alveolar Bone Status: Abnormal; Partial dentine bridge formation (0.5 e1 mm thickness). 3 (Minor Pathological changes present)—PDL: Widened PDL; Periapical Region: Minor external root resorption; Root and Alveolar Bone Status: External changes; Initial dentine bridge formation (<0.5 mm thickness). 4 (Major Pathological changes present)—PDL: Widened PDL; Periapical Region: Radiolucency present; Root and Alveolar Bone Status: Radiolucency present (No dentin bridge formation).
Bakhtiar et al. (2017) ^1^ [97]	Clinical tests and electric pulp test were performed to assess pulp vitality.	Radiographs to determine any signs of periapical pathology.

* The articles were directly cited; ^1^ References referring to the same study; ^2^ References referring to the same study.

**Table 7:** Success criteria regarding the histological parameter.

Study	Histological Criteria		
	Bridge Formation *	Inflammation Degree *	Other Characteristics *
Bakhtiar et al. (2018) [106] (Permanent t.)	Intensity of pulp inflammation: absent; mild; moderate; severe. Type of pulp inflammation: no inflammation; chronic; chronic and acute; acute. Extension pulp inflammation: absent; mild; moderate; severe.	Pulp tissue organization: normal pulp tissue; disorganization beneath the cavity; disorganization of the entire pulp tissue. Dentinal bridge morphology: complete dentinal bridge; discontinuous bridge; no signs of mineralization. Dentinal bridge thickness: more than 0.25 mm; between 0.1–0.25 mm; less than 0.1 mm.	n/a
Bakhtiar et al. (2017) [97] (Permanent t.)	Dentin bridge thickness—1: >0.25 mm; 2: 0.1–0.25 mm; 3: <0.1 mm; 4: partial or absent bridge. Morphology and continuity of dentine bridge—1: formation of a complete dentinal bridge; 2: formation of discontinuous incomplete dentin bridge; 3: no sign of dentin formation.	Type of inflammation—1: no inflammation; 2: chronic; 3: acute and chronic; 4: acute. Intensity of pulp inflammation—1: absent or very few inflammatory cells; 2: mild, <10 inflammatory cells; 3: moderate, 10–25 inflammatory cells; 4: severe, >25 inflammatory cells. Extension of pulp inflammation—1: absent; 2: mild, inflammatory cells observed in part of coronal pulp; 3: moderate, inflammatory cells observed in part of coronal pulp; 4: severe, all coronal pulp is infiltrated.	Pulp tissue organization and morphology—1: normal pulp morphology; 2: disorganization of beneath the cavity; 3: disorganization of the entire pulp.
Nowicka et al. (2015) [99] (Permanent t.)	n/a	n/a	n/a
Azimi et al. (2014) [102] (Permanent t.)	Hard tissue formation: none, partial or complete. Appearance classified as resembling: tubular; atubular; presence of tunnel defects.	0: no inflammation; 1: mild inflammation; 2: moderate inflammation; 3: severe inflammation; 4: abscess formation or extended lesions not localized to the tissue beneath the material.	n/a
Oliveira et al. (2013) [88] (Deciduous t.)	n/a	n/a	n/a
Eskandarizadeh et al. (2011) [104] (Permanent t.)	Thickness of calcified bridge (TCB)/presence of calcified bridge (PCB) (%)	Pulp inflammation—1: no inflammation (WI); 2: minimal inflammation (MI) (scattered chronic inflammatory cells beneath the calcified bridge or capping area); 3: moderate inflammation (MO) (obvious number of chronic inflammatory cells without sign of necrosis); 4: severe inflammation (SE): abscess formation, necrosis and presence of polymorphonuclear cells.	n/a
Zarrabi et al. (2011) [72] (Permanent t.)	Thickness of dentinal bridge—I: <0.1 mm; II: 0.1–0.25 mm; III: >0.25 mm. Morphology of dentinal bridge—I: no tubules present; II: irregular pattern of tubules; III: regular pattern of tubules.	I: severe inflammation or abscess; II: minimal to moderate; III: no inflammation.	Odontoblast layer—I: absent; II: presence of odontoblast cells; III: palisade pattern of cells.
Accorinte et al. (2009) [101] (Permanent t.)	Hard tissue bridge—1: complete; 2: partial bridge—little communication; 3: lateral deposition of hard tissue on the walls of the cavity of pulp exposition; 4: absence.	Inflammatory response—1: no reaction; 2: inflammatory reaction; 3: abscess; 4: necrosis.	n/a
Agamy et al. (2004) [82] (Deciduous t.)	Each specimen was observed for dentin bridge formation, odontoblastic layer integrity, pulp inflammation, pulp calcification, and pulp vitality.		

* The articles were directly cited; Permanent t: Permanent teeth; Deciduous t: Deciduous teeth.

**Table 8:** Outcomes in deciduous teeth studies regarding the clinical and radiographic parameter *.

Study	Patients			Teeth		Clinical Information		Restorative Treatment		Follow-Up									
										6 Months		1 Year		2 Years		5 Years		Other	
	n	Average [Range]	Male/Female	n	Teeth Groups	Diagnosis	Procedure	Material	Timing	Clinical	X-ray	Clinical	X-ray	Clinical	X-ray	Clinical	X-ray	Clinical	X-ray
ProRoot MTA vs. Biodentine																			
Vilella-Pastor et al. (2021) [76]	68	Male 7 [4–9] Female 6.4 ± 1.3	35/33	84	Molars	Reversible pulpitis	Pulpotomy	IRM+ SSC	I	95.3 (41/43) 97.6 (40/41)	100 (43/43) 100 (41/41)	97.4 (38/39) 100 (39/39)	97.4 (38/39) 94.9 (37/39)	100 (35/35) 100 36/36	97.1 (34/35) 94.4 (34/36)	n/a		18M 100 (37/37) 100 (38/38)	18M 94.6 (35/37) 100 (38/38)
Çelik et al. (2019) [17]	38	6.7 [5–9]	19/19	44	Mandibular Molars	Reversible pulpitis	Pulpotomy	SSC	MTA 24H BD 12M	100 (24/24) 100 (19/19)		100 (23/23) 89.5 (17/19)		100 (22/22) 89.5 (17/19)		n/a		n/a	
Bani et al. (2017) [16]	32	6.3 [4–9]	15/17	64	Mandibular Molars	Reversible pulpitis	Pulpotomy	SSC	I	100 (32/32) 100 (32/32)		96.9 (31/32) 96.9 (31/32)		96.8 (30/31) 96.8 (30/31)	87.1 (27/31) 93.5 (29/31)	n/a		n/a	
Guven et al. (2017) [77]	n/a	n/a	n/a	58	Molars	Reversible pulpitis	Pulpotomy	A	MTA 24H BD 12M	100 (29/29) 100 (29/29)	100 (29/29) 100 (29/29)	96.6 (28/29) 100 (29/29)	93.1 (27/29) 89.7 (26/29)	100 (28/28) 89.7 (26/29)	86.2 (25/29) 82.8 (24/29)	n/a		n/a	
Rajasekharan et al. (2017) [78]	38	MTA 4.65 BD 5.18	11/43	54	Maxillary 1st Molar (3) 2nd Molar (15) Mandibular 1st Molar (12) 2nd Molar (24)	Reversible pulpitis	Pulpotomy	GIC+ SSC	I	100 (29/29) 96.0 (24/25)		100 n/a 96.0 n/a	92.0 n/a 96.0 n/a	n/a		n/a		n/a	
Kusum et al. (2015) [79]	n/a	MTA 6.48 BD 6.92	n/a	50	Molars	Reversible pulpitis	Pulpotomy	ZOE +GIC SSC	1D	100 (25/25) 100 (25/25)	92.0 (23/25) 88.0 (22/25)	n/a		n/a		n/a		n/a	
ProRoot MTA vs. CEM																			
Ghajari et al. (2013) [74]	21	6.9 [5–8]	5/16	42	Maxillary 2nd Molar (19) Mandibular 2nd Molar (23)	Reversible pulpitis	Direct pulp capping	A	I	n/a		n/a		n/a		n/a		20 M 94.7 (18/19) 89.5 (17/19)	
Malekafzali et al. (2011) [80]	50	6 [4–8]	23/17	80	Maxillary 1st Molar (15) 2nd Molar (6) Mandibular 1st Molar (28) 2nd Molar (31)	Reversible pulpitis	Pulpotomy	A SSC	I	100 (36/36) 100 (36/36)		100 (33/33) 100 (33/33)	90.9 (30/33) 97.0 (32/33)	91.4* (32/35) 97.1* (34/35)	91.4* (32/35) 97.1* (34/35)	n/a		n/a	
ProRoot MTA vs. MTA Plus																			
Guven et al. (2017) [77]	n/a	n/a	n/a	58	Molars	Reversible pulpitis	Pulpotomy	A	I	100 (29/29) 100 (29/29)	100 (29/29) 100 (29/29)	96.6 (28/29) 100 (29/29)	93.1 (27/29) 96.6 (28/29)	96.6 (28/29) 100 (29/29)	93.1 (27/29) 86.2 (25/29)	n/a		n/a	
ProRoot MTA vs. NeoMTA Plus																			
Alsanouni and Bawazir (2019) [81]	28	6.0 ± 1.0	13/15	80	Molars	Reversible pulpitis	Pulpotomy	ZOE +SSC	I	100 (38/38) 100 (39/39)	100 (38/38) 97.4 (38/39)	97.4 (38/39) 100 (40/40)	94.9 (37/39) 97.4 (39/40)	n/a		n/a		n/a	
ProRoot MTA vs. Angelus MTA																			
Celik et al. (2013) [20]	n/a	n/a	n/a	91	Molars	Reversible pulpitis	Pulpotomy	GIC +A	IW	n/a		n/a		97.3 97.4	95.3 90.8	n/a		n/a	
White ProRoot MTA vs. Gray ProRoot MTA																			
Agamy et al. (2004) [82]	n/a	n/a	n/a	60	Molars	Reversible pulpitis	Pulpotomy	IRM +SSC	I	95.0 (19/20) 96.0 (24/25)	n/a	80.0 (16/20) 100 (19/19)		n/a		n/a		n/a	
ProRoot MTA vs. TheraCal-LC																			
Hassanpour et al. (2023) [83]	45	[5–8]	24/21	90	Molars	Reversible pulpitis	Pulpotomy	GIC +SSC	I	100 (45/45) 97.8 (44/45)	100 (45/45) n/a	100 (41/41) 99.4 n/a	98.8 97.2	n/a		n/a		n/a	
Erfanparast et al. (2018) [84]	46	6.3 [5–7]	25/21	92	Molars	Reversible pulpitis	Direct pulp capping	IRM +A	I	97.8 (45/46) 97.8 (45/46)		94.6 (35/37) 91.9 (34/37)	100 (37/37) 100 (37/37)	n/a		n/a		n/a	
ProRoot MTA vs. OrthoMTA																			
Kang et al. (2015) [85]	143	n/a [3–10]	60/42	105	Molars	Reversible pulpitis	Pulpotomy	Caviton RMGIC +SSC	3W	100 (38/38) 93.8 (30/32)	100 (38/38) 97.6 (40/41)	100 (38/38) 97.4 (37/38)	100 (33/33) 94.7 (36/38)	n/a		n/a		n/a	
ProRoot MTA vs. RetroMTA																			
Kang et al. (2015) [85]	143	n/a [3–10]	60/42	105	Molars	Reversible pulpitis	Pulpotomy	MTA Caviton RMGIC +SSC Retro RMGIC +SSC	MTA 3W Retro I	100 (38/38) 100 (46/46)	100 (38/38) 93.5 (43/46)	100 (38/38) 100 (38/38)	100 (33/33) 94.7 (36/38)	n/a		n/a		n/a	
ProRoot MTA vs. Endocem																			
Joo et al. (2023) [86]	n/a	n/a	n/a	107	Molars	Reversible pulpitis	Pulpotomy	RGIC/GIC + SSC /ZC/3D C	I	(49/50) (51/53)	(49/50) (48/53)	(45/46) (45/47)	(44/46) (40/47)					3M (50/50) (53/53)	3M (49/50) (48/53)
ProRoot MTA vs. BC Putty																			
Joo et al. (2023) [86]	n/a	n/a	n/a	107	Molars	Reversible pulpitis	Pulpotomy	RGIC/GIC + SSC /ZC/3D C	I	(49/50) (50/50)	(49/50) (47/50)	(45/46) (46/46)	(44/46) (42/46)					3M (50/50) (50/50)	3M (49/50) (50/50)
ProRoot MTA vs. Portland																			
Yildirim et al. (2016) [87]	n/a	n/a	n/a	70	1st Molar (33) 2nd Molar (37)	Reversible pulpitis	Pulpotomy	GIC +SSC	I	100 (34/34) 93.9 (31/33)	n/a	100 (33/33) 93.9 (29/31)	n/a	100 (33/33) 93.3 (28/30)	93.9 (31/33) 86.7 (26/30)	n/a		n/a	
OrthoMTA vs. RetroMTA																			
Kang et al. (2015) [85]	143	n/a [3–10]	60/42	105	Molars	Reversible pulpitis	Pulpotomy	MTA Caviton RMGIC +SSC Retro RMGIC +SSC	MTA 3W Ortho I	100 (41/41) 100 (46/46)	97.6 (40/41) 93.5 (43/46)	97.4 (37/38) 100 (38/38)	94.7 (36/38) 94.7 (36/38)	n/a		n/a		n/a	
MTA Plus vs. Biodentine																			
Guven et al. (2017) [77]	n/a	n/a	n/a	58	Molars	Reversible pulpitis	Pulpotomy	A	MTA I BD 12M	100 (29/29) 100 (29/29)	100 (29/29) 100 (29/29)	100 (29/29) 100 (29/29)	96.6 (28/29) 89.7 (26/29)	100 (29/29) 100 (29/29)	86.2 (25/29) 82.8 (24/29)	n/a		n/a	
NeoMTA vs. NeoPUTTY																			
Alqahtani et al. (2023) [88]	42	6.3 ± 1.4 [4–9]	23/19	70	Molars	Reversible pulpitis	Pulpotomy	RGIC +SSC	I	100 (35/35) 100 (35/35)	94.3 (33/35) 94.3 (33/35)	100 (34/34) 97.1 (34/35)	94.1 (33/34) 91.4 (32/35)	n/a		n/a		n/a	
Angelus MTA vs. Portland																			
Oliveira et al. (2013) [89]	n/a	n/a	n/a	30	Mandibular Molars	Reversible pulpitis	Pulpotomy	ZOE+ RMGIC	I	100 (15/15) 100 (15/15)		100 (15/15) 100 (15/15)		100 (15/15) 100 (15/15)		n/a		n/a	
Sakai et al. (2009) [90]	30	6.9 [5–9]	19/11	30	Mandibular Molars	Reversible pulpitis	Pulpotomy	IRM+ RMGIC	I	100 (14/14) 100 (15/15)		100 (13/13) 100 (15/15)		100 (9/9) 100 (9/9)		n/a		n/a	
Angelus MTA vs. Bio-C Pulpo																			
Lima et al. (2020) [91]	33	5.7 ± 1.6	18/15	70	Molars	Reversible pulpitis	Pulpotomy	C	I	100 (32/32) 100 (33/33)	96.9 (31/32) 100 (33/33)	n/a		n/a		n/a		n/a	
Angelus MTA vs. Biodentine																			
Ramanandvignesh et al. (2020) [92]	n/a	[4–9]	n/a	36	Mandibular 1st Molar	Reversible pulpitis	Pulpotomy	ZOE+ Cermet /SSC	24H	82.3 (14/17) 100 (17/17)	82.3 (14/17) 94.1 (16/17)	n/a		n/a		n/a		9M 82.3 (14/17) 100 (17/17)	9M 82.3 (14/17) 94.1 (16/17)
Angelus MTA vs. BC putty																			
Alnassar et al. (2022) [93]	40	6.9 ± 0.75 [6–8]	19/21	40	Mandibular Molars	Irreversible pulpitis	Pulpotomy	GIC +SSC	I	95.0 (19/20) 100 (20/20)	95.0 (19/20) 100 (20/20)	95.0 (19/20) 100 (20/20)	95.0 (19/20) 100 (20/20)	n/a		n/a		n/a	

Calcium silicates cements: ProRoot MTA (ProRoot^®^—MTA Dentsply Tulsa Dental, Johnson City, TN, USA); Biodentine/BD (Biodentine^TM^—Septodont, Saint-Maur-des-Fossés Cedex, France); CEM (CEM—Bionique Dent, Tehran, Iran); MTA Plus (MTA Plus^®^ Avalon Biomed Inc., Houston, Texas); NeoMTA Plus (NeoMTA Plus^®^—Avalon Biomed Inc., Bradenton, Fla., USA); NeoMTA (NeoMTA 2 NuSmile Inc., Houston, Texas, USA); NeoPUTTY (NeoPUTTY^®^—NuSmile Inc., Houston, Texas, USA); MTA Angelus (Angelus^®^ MTA Londrina, Paraná, Brazil); W MTA (White ProRoot^®^ MTA—Dentsply Tulsa Dental, Johnson City, TN, USA); G MTA (Gray MTA ProRoot^®^ MTA—Dentsply Tulsa Dental, Johnson City, TN, USA); Portland (Portland cement Votorantim—Cimentos, São Paulo, SP, Brazil); Theracal (TheraCal-LC^®—^Bisco Inc., Schamburg, IL, USA); OrthoMTA/Ortho (OrthoMTA^®^ BioMTA, Seoul, Republic of Korea); Retro MTA/Retro (RetroMTA^®^ BioMTA, Seoul, Republic of Korea); Bio-C Pulpo (Bio-C^®^ Pulpo—Angelus, Londrina, PR, Brazil); BC Putty (Well-Root^TM^ PT Bioceramic putty (Vericom, Republic of Korea); Endocem (Endocem—Maruchi Regenerative Endodontic materials). Coronal restoration: A (Amalgam); C (Composite); SSC (Stainless steel crown); GIC (Glass ionomer cement); ZD (prefabricated zirconia crowns—PZC; NuSmile Zr Zirconia^®^, Houston, Texas, USA); 3D C (3D-printed resin crowns—3DRC; Tera Harz C&B 80DP, Graphy, Seoul, Republic of Korea, and Sprintray 3D printer, US); RMGIC (Resin modified glass ionomer cement); IRM (IRM^®^ Intermediate Restorative Material—Dentsply Sirona); Caviton (Caviton GC, Tokyo, Japan. Timing: I (Immediate); M (Minute); H (Hours); D (Day); W (Week). n/a: not available. * only considered the overall analysis.

**Table 9:** Outcomes regarding the histological parameter for both permanent and deciduous dentitions.

Study	Follow-Up	Histology Results *			Conclusions *
		Bridge Formation	Inflammation Degree	Other Characteristics	
ProRoot MTA vs. Biodentine					
Bakhtiar et al. (2017) [97] (Permanent t.)	8W	Dentinal bridge morphology and continuity—formation of hard tissue beneath the cavity in the form of complete dentinal bridge = 5; formation of discontinuous bridge beneath the cavity (incomplete dentinal bridge) = 4; no signs of dentin formation = 0. Dentinal bridge thickness—more than 0.25 mm = 1; between 0.1 and 0.25 mm = 8; less than 0.1 mm = 0.	Intensity of pulp inflammation—absent = 9; type of pulp inflammation—absent = 9; extension of pulp inflammation—absent = 9.	Pulp tissue organization and morphology—normal or almost normal pulp tissue morphology = 3; disorganization of pulp tissue beneath the cavity = 2; disorganization of entire pulp tissue = 4.	Dentin bridge at the site of injury was uniform and homogenous with Biodentine, followed by ProRoot MTA.
		Dentinal bridge morphology and continuity—formation of hard tissue beneath the cavity in the form of complete dentinal bridge = 9; formation of discontinuous bridge beneath the cavity (incomplete dentinal bridge) = 0; no signs of dentin formation (0). Dentinal bridge thickness—more than 0.25 mm = 5; Between 0.1 and 0.25 mm = 4; Less than 0.1 mm = 0.	Intensity of pulp inflammation—absent = 8; mild = 1. Type of pulp inflammation—absent = 8; mild = 1. Extension of pulp inflammation—absent = 8; mild = 1.	Pulp tissue organization and morphology—normal or almost normal pulp tissue morphology = 6; disorganization of pulp tissue beneath the cavity = 3; disorganization of entire pulp tissue = 0.	
Nowicka et al. (2015) [99] (Permanent t.)	6W	MTA and Biodentine actively initiated the formation of reparative dentin in each tooth. Impossible to quantify from the graphic presented.	n/a	n/a	MTA and Biodentine actively initiated the formation of reparative dentin in each tooth (n = 11). The thickness of the dentin bridges displayed no significant different between the MTA and Biodentine groups.
ProRoot MTA vs. TheraCal-LC					
Bakhtiar et al. (2017) [97] (Permanent t.)	8W	Dentinal bridge morphology and continuity—formation of hard tissue beneath the cavity in the form of complete dentinal bridge = 5; formation of discontinuous bridge beneath the cavity (incomplete dentinal bridge) = 4; no signs of dentin formation = 0. Dentinal bridge thickness—more than 0.25 mm = 1; between 0.1 and 0.25 mm = 8; less than 0.1 mm = 0.	Intensity of pulp inflammation—absent = 9. Type of pulp inflammation—absent = 9. Extension of pulp inflammation—absent = 9.	Pulp tissue organization and morphology—normal or almost normal pulp tissue morphology = 3; disorganization of pulp tissue beneath the cavity = 2; disorganization of entire pulp tissue = 4.	Normal pulp organization was seen in 33.33% of the teeth in ProRoot MTA, and in 11.11% of the TheraCal group (*p* = 0.06). Complete dentinal bridge formation rate was 11% and 56% in TheraCal and ProRoot MTA groups, respectively.
		Dentinal bridge morphology and continuity—formation of hard tissue beneath the cavity in the form of complete dentinal bridge = 1; formation of discontinuous bridge beneath the cavity (incomplete dentinal bridge) = 6; no signs of dentin formation = 2. Dentinal bridge thickness—more than 0.25 mm = 5; between 0.1 and 0.25 mm = 2; less than 0.1 mm = 2.	Intensity of pulp inflammation—absent = 9. Type of pulp—absent = 9. Extension of pulp inflammation—absent = 9.	Pulp tissue organization and morphology—normal or almost normal pulp tissue morphology = 1; disorganization of pulp tissue beneath the cavity = 6; disorganization of entire pulp tissue = 2.	
Biodentine vs. TheraCal-LC					
Bakhtiar et al. (2017) [97] (Permanent t.)	8W	Dentinal bridge morphology and continuity—formation of hard tissue beneath the cavity in the form of complete dentinal bridge = 9; formation of discontinuous bridge beneath the cavity (incomplete dentinal bridge) = 0; no signs of dentin formation = 0. Dentinal bridge thickness—more than 0.25 mm = 5; between 0.1 and 0.25 mm = 4; less than 0.1 mm = 0.	Intensity of pulp inflammation—absent = 8; mild = 1. Type of pulp inflammation—absent = 8; mild = 1. Extension of pulp inflammation—absent = 8; mild = 1.	Pulp tissue organization and morphology—normal or almost normal pulp tissue morphology = 6; disorganization of pulp tissue beneath the cavity = 3; disorganization of entire pulp tissue = 0.	Normal pulp organization was seen in 11.11% of the TheraCal-LC group and in 66.67% of the Biodentine group (*p* = 0.06). The Biodentine group showed complete dentinal bridge formation in all teeth, whereas this rate was 11% in the TheraCal-LC group.
		Dentinal bridge morphology and continuity—formation of hard tissue beneath the cavity in the form of complete dentinal bridge = 1; formation of discontinuous bridge beneath the cavity (incomplete dentinal bridge) = 6; no signs of dentin formation = 2. Dentinal bridge thickness—none than 0.25 mm = 5; between 0.1 and 0.25 mm = 2; less than 0.1 mm = 2.	Intensity of pulp inflammation—absent = 9. Type of pulp—absent = 9. Extension of pulp inflammation—absent = 9.	Pulp tissue organization and morphology—normal or almost normal pulp tissue morphology = 1; disorganization of pulp tissue beneath the cavity = 6; disorganization of entire pulp tissue = 2.	
ProRoot MTA vs. iRoot BP					
Azimi et al. (2014) [102] (Permanent t.)	6W	Hard tissue formation: none = 0; partial = 4; complete = 8. Appearance classified as resembling: tubular = 2; atubular = 8; presence of tunnel defects = 2.	0 =0; 1 = 7; 2 = 4; 3 = 1; 4 = 0.	n/a	No significant difference between ProRoot MTA and I Root BP in terms of pulp inflammation; formation of hard tissue bridge and its appearance was detected.
		Hard tissue formation: none = 0; partial = 5; complete = 7. Appearance classified as resembling: tubular = 3; atubular = 8; presence of tunnel defects = 1.	0 = 0; 1 = 8; 2 = 3; 3 = 1; 4 = 0.	n/a	
ProRoot MTA vs. CEM					
Zarrabi et al. (2011) [72] (Permanent t.)	2W	Morphology of dentinal bridge—I = 3; II = 5; III = 0. Thickness—I = 5; II = 3; III = 0.	Intensity of pulp inflammation—I = 3; II = 5; III = 0.	Odontoblast layer—I = 2; II = 6; III = 0.	Both MTA and CEM showed significantly better pulp response after 8 weeks compared with 2 weeks, with a thicker and more tubular pattern of the dentinal bridge, less pulp inflammation, and a palisade pattern of odontoblast cells. Although MTA and CEM groups had no significant difference in each measure in both time intervals, CEM induced a thicker dentinal bridge with less pulp inflammation at both 2 weeks and 8 weeks, compared with MTA.
		Morphology of dentinal bridge—I = 5; II = 3; III = 0. Thickness—I = 4; II = 4; III = 0.	Intensity of pulp inflammation—I = 0; II = 1; III = 7.	Odontoblast layer—I = 2; II = 6; III = 0.	
	8W	Morphology of dentinal bridge—I = 0; II = 6; III = 2. Thickness—I = 0; II = 5; III = 3.	Intensity of pulp inflammation —I = 0; II = 6; III = 2.	Odontoblast layer—I = 0; II = 4; III = 4.	
		Morphology of dentinal bridge—I = 0; II = 4; III = 4. Thickness—I =0; II = 2; III = 6.	Intensity of pulp inflammation—I = 0; II = 1; III = 7.	Odontoblast layer—I = 0; II = 5; III = 3.	
White MTA ProRoot vs. Gray MTA ProRoot					
Agamy et al. (2004) [82] (Deciduous t.)		Not quantified	Not quantified	Odontoblastic layer integrity, pulp calcification, and pulp vitality.	In the histologic study, both types of MTA successfully induced thick dentin bridge formation at the amputation sites. Teeth treated with gray MTA demonstrated pulp architecture nearest to normal pulp by preserving the odontoblastic layer and delicate fibrocellular matrix, yet few inflammatory cells or isolated calcified bodies were seen. Teeth treated with white MTA showed a denser fibrotic pattern, with more isolated calcifications in the pulp tissue along with secondary dentin formation.
Eskandarizadeh et al. (2011) [104] (Permanent t.)	30D	Thickness of calcified bridge: 188 ± 113. Presence of calcified bridge (%): 10(100).	Pulp inflammation—no inflammation = 50%; minimal inflammation = 50%.	n/a	Most WMTA specimens and all GMTA specimens showed either free of inflammation or minor pulpal inflammation at 60- and 90-day intervals. GMTA specimens showed no significant difference to WMTA in terms of the presence and thickness of the calcified bridge, as well as the severity of pulp inflammatory response, to either of the pulp capping materials at all time intervals of the present study (*p* > 0.05).
		Thickness of calcified bridge: 134 ± 21. Presence of calcified bridge: 9(90).	no inflammation = 40%; minimal inflammation = 40%; moderate inflammation = 20%.	n/a	
	60D	Thickness of calcified bridge: 191 ± 105. Presence of calcified bridge: 10(100).	no inflammation = 60%; minimal inflammation = 30%; moderate inflammation = 10%.	n/a	
		Thickness of calcified bridge: 275 ± 67. Presence of calcified bridge: 10(100).	no inflammation = 60%; minimal inflammation = 40%.	n/a	
	90D	Thickness of calcified bridge: 330 ± 196. Presence of calcified bridge: 10(100)	no inflammation = 40%; minimal inflammation = 60%.	n/a	
		Thickness of calcified bridge: 264 ± 85. Presence of calcified bridge: 10(100).	no inflammation = 70%; minimal inflammation = 30%.	n/a	
Gray MTA ProRoot vs. Gray Angelus MTA					
Accorinte et al. (2009) [101] (Permanent t.)	30D	Hard tissue bridge—complete (1) = 5; 2: partial bridge—little communication (2) = 1; lateral deposition of hard tissue on the walls of the cavity of pulp exposition (3) = 1; absence (4) = 1.	Inflammatory response—no reaction (1) = 2; inflammatory reaction (2) = 6.	Giant cells—absent (1) = 8; great (4) = 1. Particles of the capping material—absent (1) = 6; mild number (2) = 1; moderate (3) = 1.	No significant difference was observed between the two materials (*p* > 0.05) in terms of overall histological features (hard tissue bridge, inflammatory response, giant cells, and particles of capping materials). Overall, 94% and 88% of the specimens capped with Angelus MTA and ProRoot MTA, respectively, showed either total or partial hard tissue bridge formation (*p* > 0.05).
		Hard tissue bridge—1 = 5; 3 = 2; 4 = 1.	Inflammatory response—1 = 3; 2 = 4; abscess (3) = 1.	Giant cells—1 = 7; moderate (3) = 1. Particles of the capping material—1 = 7; 3 = 1.	
	60D	Hard tissue bridge—1 = 5; 3 = 1; 4 = 1.	1 = 3; 2 = 5; necrosis (4) =1.	Giant cells—1 = 8; 4 = 1. Particles of the capping material—1 = 8; Great (4) = 1.	
		Hard tissue bridge—1 = 6; 2 = 1; 3 = 3.	Inflammatory response—1 = 7; 2 = 3.	Giant cells—1 = 9; mild number (2) = 1. Particles of the capping material—1 = 9; mild number (2) = 1.	
Angelus MTA vs. Portland					
Oliveira et al. (2013) [88] (Deciduous t.)	Histological findings revealed the presence of dentine-like mineralized material deposition obliterating the root canal and some dentine barrier formation in the Portland Cement and MTA groups.				
ProRoot MTA vs. Retro MTA					
Bakhtiar et al. (2018) [106] (Permanent t.)	8W	Pulp tissue organization—normal pulp tissue = 6; disorganization beneath the cavity = 2. Disorganization of the entire pulp tissue = 3. Dentinal bridge morphology—complete dentinal bridge = 7; discontinuous bridge = 4. Dentinal bridge thickness—more than 0.25 mm = 5; between 0.1–0.25 mm = 6.	Intensity of pulp inflammation—absent = 11. Type of pulp inflammation—no Inflammation = 11. Extension pulp inflammation—absent = 11.	n/a	In the Retro MTA group, this study revealed the formation of a discontinuous bridge in most cases under the material within the pulp tissue, with no significant inflammatory reaction in partially or completely disorganized dental pulp. This contrasts with ProRoot MTA, which resulted in complete dentin bridge formation in most of the teeth with no inflammation and normal pulp morphology.
		Pulp tissue organization—normal pulp tissue = 1; disorganization beneath the cavity = 3; disorganization of the entire pulp tissue = 7. Dentinal bridge morphology—complete dentinal bridge = 3; discontinuous bridge = 7; no signs of mineralization = 1. Dentinal bridge thickness—between 0.1–0.25 mm = 5; less than 0.1 mm = 6.	Intensity of pulp inflammation—absent = 8; mild = 3.Type of pulp inflammation—no Inflammation = 8; chronic = 3. Extension pulp inflammation—absent = 8; moderate =3.	n/a	

n/a: not available. * The articles were directly cited; Permanent t: Permanent teeth; Deciduous t: Deciduous teeth; D, Days; W, Weeks.

**Table 10:** Outcomes in permanent teeth studies regarding the clinical and radiographic parameters.

Study	Patients			Teeth		Clinical Information		Restorative Treatment		Follow-Up									
										6 Months		1 Year		2 Years		5 Years		Other	
	n	Average [Range]	Male/ Female	n	Teeth Groups	Diagnosis	Procedure	Material	Timing	Clinical	X-ray	Clinical	X-ray	Clinical	X-ray	Clinical	X-ray	Clinical	X-ray
ProRoot MTA vs. Biodentine																			
Singh et al. (2023) [94]	49	n/a	22/27	49	Molars	Reversible pulpitis	Pulpotomy	GIC +C	1W	n/a		91.7 (22/24) 100 (22/22)		n/a		n/a		n/a	
Taha et al. (2022) [95]	n/a	n/a	n/a	100	Molars	Irreversible and Reversible pulpitis	Pulpotomy	RMGIC +C	I	92.7 (38/41) 90.9 (30/33)		97.8 (45/46) 100 (42/42)		n/a		n/a		n/a	
Uyar and Alaçam (2021) [96]	n/a	n/a	n/a	36	Immature molars	Reversible pulpitis	Pulpotomy	GIC +SSC	I	100 (18/18) 100 (18/18)		94.4 (17/18) 94.4 (17/18)						3M 100 (18/18) 100 (18/18)	
Bakhtiar et al. (2017) [97]	27	[18–32]	n/a	27	Maxillary 3rd Molar Mandibular 3rd Molar	Normal pulp	Pulpotomy	GIC	I	n/a		n/a		n/a		n/a		2M 100 n/a 100 n/a	
Brizuela et al. (2017) [98]	116	11.51 11.22	56/60	116	Maxillary 1st Molar (33) 2nd Molar (2) Mandibular 1st Molar (68) 2nd Molar (13)	Normal pulp Reversible pulpitis	Direct pulp capping	C	I	91.9 (34/37) 100 (38/38		100 (22/22) 100 (25/25)		n/a		n/a		n/a	
Nowicka et al. (2015) [99]	n/a	n/a	n/a	22	Maxillary 3rd Molar Mandibular 3rd Molar	Normal pulp	Direct pulp capping	MTA GIC, C BD C	1W	n/a		n/a		n/a		n/a		1.5M 100 (11/11) 100 (11/11)	
ProRoot MTA vs. Angelus MTA																			
Katge and Patil (2017) [100]	29	n/a	n/a	58	Molars	Reversible pulpitis	Direct pulp capping	MTA RMGICC BD C	3M	100 (21/21) 100 (21/21)		100 (21/21) 100 (21/21)		n/a		n/a		n/a	
Gray MTA ProRoot vs. Gray Angelus MTA																			
Accorinte et al. (2009) [101]	n/a	[25–42]	n/a	40	Premolar	Normal pulp	Direct pulp capping	ZOE	I	Histological outcomes at 30 and 60 days									
ProRoot MTA vs. iRoot BP																			
Azimi et al. (2014) [102]	12	14 [12–16]	n/a	24	1st Premolar	Normal pulp	Pulpotomy	RMGIC C	I	n/a		n/a		n/a		n/a		1.5M 100 (12/12) 100 (12/12)	
ProRoot MTA vs. CEM																			
Asgary et al. (2022) [103]	n/a	[10–60]	35/71	106	Molars	Irreversible pulpitis	Pulpotomy	C	I	n/a		n/a		100 51/51 100 51/51	100 47/47 97,9 46/47	n/a	n/a	n/a	
Asgary et al. (2017) [73]	412	[9–65]	n/a	412	1st Molar 2nd Molar	Irreversible pulpitis	Pulpotomy	Cavit+A	1W	n/a		n/a		98.9 (176/178 96.4 (163/169	94.9 (169/178 86.1 (143/166	98.1 (151/154) 98.0 (147/150)	84.7 (116/137) 78.1 (107/137)	n/a	
White ProRoot MTA vs. Gray ProRoot MTA																			
Eskandarizadeh et al. (2011) [104]	n/a			60	1st Premolar 2nd Premolar	Normal pulp	Direct pulp capping	A	I	Histological outcomes at 30, 60, and 90 days									
ProRoot MTA vs. OrthoMTA																			
Kang et al. (2017) [105]	82	29.3	31/51	104	Premolar Molar	Reversible pulpitis	Pulpotomy	IRM, RMGIC+ C/Cer	2-3D	96.4 (27/28) 93.8 (30/32)		96.0 (24/25) 92.8 (26/28)		n/a		n/a		n/a	
ProRoot MTA vs. RetroMTA																			
Kang et al. (2017) [105]	82	29.3	31/51	104	Premolar Molar	Reversible pulpitis	Pulpotomy	MTA IRM, RMGIC +C/Cer Retro RMGIC +C/Cer	MTA 2-3D Retro I	96.4 (27/28) 96.8 (30/31)		96.0 (24/25) 96.2 (25/26)		n/a		n/a		n/a	
Bakhtiar et al. (2018) [106]	n/a	[18–32]	n/a	22	3rd Molar	Normal pulp	Pulpotomy	GIC	I	Histological outcomes at 8 weeks									
ProRoot MTA vs. TotalFill																			
Taha et al. (2022) [95]	n/a	n/a	n/a	114	Molars	Irreversible and Reversible pulpitis	Pulpotomy	RMGIC +C	I	92.7 (38/41) 92.6 (50/54)		97.8 (45/46) 100 (58/58)		n/a		n/a		n/a	
ProRoot MTA vs. Endocem																			
Jang et al. (2015) [107]	35	72 [19–79]	n/a	46	Incisor + Premolar (24) Molar (22)	Reversible pulpitis	Direct pulp capping	C, Cer	3M	n/a		87.0 (20/23) 83.3 15/18		n/a		n/a		n/a	
ProRoot MTA vs. TheraCal-LC																			
Bakhtiar et al. (2017) [97]	27	[18–32]	n/a	27	3rd Molar	Normal pulp	Pulpotomy	GIC	I	n/a		n/a		n/a		n/a		2M 100 n/a 100 n/a	
Angelus MTA vs. Biodentine																			
Awawdeh et al. (2018) [108]	58	n/a [16–59]	23/35	68	Incisor (5) Premolar (18) Molar (45)	Reversible pulpitis	Direct pulp capping (17) Pulpotomy (51)	MTA IRM/ A, C BD A, C	1W	93.5 (29/31) 93.1 (27/29)		100 (28/28) 96.0 (24/25)		100 (27/27) 100 (24/24)		n/a		3Y 96.0 (24/25) 91.7 (22/24)	
OrthoMTA vs. Retro MTA																			
Kang et al. (2017) [105]	82	29.3	31/51	104	Premolar Molar	Reversible pulpitis	Pulpotomy	MTA IRM/ RMGIC +C, Cer Retro RMGIC +C, Cer	MTA 2-3D Ortho I	93.8 (30/32) 96.8 (30/31)		92.9 (26/28) 96.2 (25/26)		n/a		n/a		n/a	
Angelus MTA vs. CEM																			
Zarrabi et al. (2011) [72]	n/a	[15–25]	n/a	32	1st Premolar	Normal pulp	Direct pulp capping	GIC+C	I	n/a		n/a		n/a		n/a		n/a (histology)	
MTA+ vs. Biodentine																			
Peskersoy et al. (2021) [109]	n/a	n/a	n/a	210	Molars	Reversible pulpitis	Direct pulp capping	C	I	86.0 n/a 84.0 n/a	88.0 n/a 86.0 n/a	86.0 n/a 80.0 n/a	86.0 n/a 82.0 n/a	n/a		n/a		3Y 85 n/a 79 n/a	3Y 86 n/a 80 n/a
MTA+ vs. TheraCal-LC																			
Peskersoy et al. (2021) [109]	n/a	n/a	n/a	210	Molars	Reversible pulpitis	Direct pulp capping	C	I	86 n/a 83 n/a	88.0 n/a 81.0 n/a	86.0 n/a 73.0 n/a	86.0 n/a 73.0 n/a	n/a		n/a		3Y 85 n/a 72 n/a	3Y 86 n/a 73 n/a
Biodentine vs. TotalFill																			
Taha et al. (2022) [95]	n/a	n/a	n/a	114	Molars	Irreversible and Reversible pulpitis	Pulpotomy	RMGIC +C	I	90.9 (30/33) 92.6 (50/54)		100 (42/42) 100 (58/58)		n/a		n/a		n/a	
Biodentine vs. TheraCal-LC																			
Peskersoy et al. (2021) [109]	n/a	n/a	n/a	210	Molars	Reversible pulpitis	Direct pulp capping	C	I	84.0 n/a 83.0 n/a	86.0 n/a 81.0 n/a	80.0 n/a 73.0 n/a	82.0 n/a 73.0 n/a	n/a		n/a		3Y 79 n/a 72 n/a	3Y 80 n/a 73 n/a
Bakhtiar et al. (2017) [97]	27	[18–32]	n/a	27	3rd Molar	Normal pulp	Pulpotomy	GIC	I	n/a		n/a		n/a		n/a		2M 100 n/a 100 n/a	

Calcium silicates cements: ProRoot MTA/MTA (ProRoot^®^ MTA (Dentsply Tulsa Dental, Johnson City, TN, USA); Biodentine/BD (Biodentine^TM^—Septodont, Saint-Maur-des-Fossés Cedex, France); CEM (CEM—Bionique Dent, Tehran, Iran); MTA-Plus (MTA Plus^®^ Avalon Biomed Inc., Houston, Texas): MTA Angelus (Angelus^®^ MTA—Londrina, Paraná, Brazil); W MTA (White ProRoot^®^ MTA—Dentsply Tulsa Dental, Johnson City, TN, USA); G MTA (Gray MTA ProRoot^®^ MTA (Dentsply Tulsa Dental, Johnson City, TN, USA); Theracal (TheraCal-LC^®^—Bisco Inc., Schamburg, IL, USA); OrthoMTA/Ortho (OrthoMTA—BioMTA, Seoul, Republic of Korea); RetroMTA/Retro (RetroMTA—BioMTA, Seoul, Republic of Korea); iRoot BP (iRoot BP—Innovative Bio Ceramix, Inc., Vancouver, BC, Canada); Endocem (Endocem—Maruchi Regenerative Endodontic materials); MTA+ (MTA PlusTM– Prevest DentPro^®^, Avalon Biomed Inc. Bradenton, FL, USA); TotalFill/TF (TotalFill BC RRM Fast Set Putty—FKG). Coronal restoration: A (Amalgam); C (Composite); SSC (Stainless steel crown); GIC (Glass ionomer cement); RMGIC (Resin modified glass ionomer cement); Cavit (3MTM CAVITTM Temporary Filling Material—3M ESPE AG); Cer (Ceramic); IRM (IRM^®^ Intermediate Restorative Material—Dentsply DeTrey GmbH). Timing: I (Immediate); H (Hours); D (Day); W (Week); n/a: not available.

## Data Availability

Data are contained within the article.
